# Combining cisplatin and a STING agonist into one molecule for metalloimmunotherapy of cancer

**DOI:** 10.1093/nsr/nwae020

**Published:** 2024-01-17

**Authors:** Shuren Zhang, Dongfan Song, Wenhao Yu, Ji Li, Xiaoyu Wang, Yachao Li, Zihan Zhao, Qi Xue, Jing Zhao, Jie P Li, Zijian Guo

**Affiliations:** State Key Laboratory of Coordination Chemistry, School of Chemistry and Chemical Engineering, Chemistry and Biomedicine Innovation Center (ChemBIC), Nanjing University, Nanjing 210023, China; State Key Laboratory of Coordination Chemistry, School of Chemistry and Chemical Engineering, Chemistry and Biomedicine Innovation Center (ChemBIC), Nanjing University, Nanjing 210023, China; State Key Laboratory of Coordination Chemistry, School of Chemistry and Chemical Engineering, Chemistry and Biomedicine Innovation Center (ChemBIC), Nanjing University, Nanjing 210023, China; State Key Laboratory of Coordination Chemistry, School of Chemistry and Chemical Engineering, Chemistry and Biomedicine Innovation Center (ChemBIC), Nanjing University, Nanjing 210023, China; State Key Laboratory of Coordination Chemistry, School of Chemistry and Chemical Engineering, Chemistry and Biomedicine Innovation Center (ChemBIC), Nanjing University, Nanjing 210023, China; State Key Laboratory of Coordination Chemistry, School of Chemistry and Chemical Engineering, Chemistry and Biomedicine Innovation Center (ChemBIC), Nanjing University, Nanjing 210023, China; Department of Urology, Affiliated Drum Tower Hospital, Medical School, Nanjing University, Nanjing 210023, China; State Key Laboratory of Coordination Chemistry, School of Chemistry and Chemical Engineering, Chemistry and Biomedicine Innovation Center (ChemBIC), Nanjing University, Nanjing 210023, China; State Key Laboratory of Coordination Chemistry, School of Chemistry and Chemical Engineering, Chemistry and Biomedicine Innovation Center (ChemBIC), Nanjing University, Nanjing 210023, China; Nanchuang (Jiangsu) Institute of Chemistry and Health, Nanjing 210023, China; State Key Laboratory of Coordination Chemistry, School of Chemistry and Chemical Engineering, Chemistry and Biomedicine Innovation Center (ChemBIC), Nanjing University, Nanjing 210023, China; State Key Laboratory of Coordination Chemistry, School of Chemistry and Chemical Engineering, Chemistry and Biomedicine Innovation Center (ChemBIC), Nanjing University, Nanjing 210023, China; Nanchuang (Jiangsu) Institute of Chemistry and Health, Nanjing 210023, China

**Keywords:** platinum drug, STING agonist, single-molecule multitarget, metalloimmunology

## Abstract

Mounting evidence suggests that strategies combining DNA-damaging agents and stimulator of interferon genes (STING) agonists are promising cancer therapeutic regimens because they can amplify STING activation and remodel the immunosuppressive tumor microenvironment. However, a single molecular entity comprising both agents has not yet been developed. Herein, we designed two Pt^IV^-MSA-2 conjugates (**I** and **II**) containing the DNA-damaging chemotherapeutic drug cisplatin and the innate immune-activating STING agonist MSA-2; these conjugates showed great potential as multispecific small-molecule drugs against pancreatic cancer. Mechanistic studies revealed that conjugate **I** upregulated the expression of transcripts associated with innate immunity and metabolism in cancer cells, significantly differing from cisplatin and MSA-2. An analysis of the tumor microenvironment demonstrated that conjugate **I** could enhance the infiltration of natural killer (NK) cells into tumors and promote the activation of T cells, NK cells and dendritic cells in tumor tissues. These findings indicated that conjugate **I**, which was created by incorporating a Pt chemotherapeutic drug and STING agonist into one molecule, is a promising and potent anticancer drug candidate, opening new avenues for small-molecule-based cancer metalloimmunotherapy.

## Teaser

A small-molecule metallo-immunotherapeutic, Pt-MSA-2-I, created by incorporating DNA damaging cisplatin and a STING agonist into one molecule, can enhance STING activation and remodel the immunosuppressive tumor microenvironment, showing potential against pancreatic cancer.

## INTRODUCTION

Emerging cancer immunotherapies (CITs) have achieved great clinical success in various tumors in the past decade, yet only a small subset of patients with certain immune-sensitive cancer types achieve life-altering durable survival with these therapies [1–6]. Currently, most CITs, such as immune checkpoint blockade (ICB) and adoptive cell transfer (ACT)-based therapies, potentiate the antitumor adaptive immune response by manipulating T cells [5]. However, immune-suppressive tumor microenvironments (TME) are usually characterized by a remarkable lack of T-cell infiltration, which is typically considered a characteristic of immunologically ‘cold’ tumors [6]. In contrast, there is frequently more infiltration of innate immune cells than T cells in many tumor tissues, and these cells can be transformed to attract and educate antigen-specific T cells [7]. Therefore, using molecules to initiate and activate innate immunity has been recognized as an attractive strategy to convert a ‘cold’ tumor microenvironment into a ‘hot’ microenvironment.

**Figure 1.**
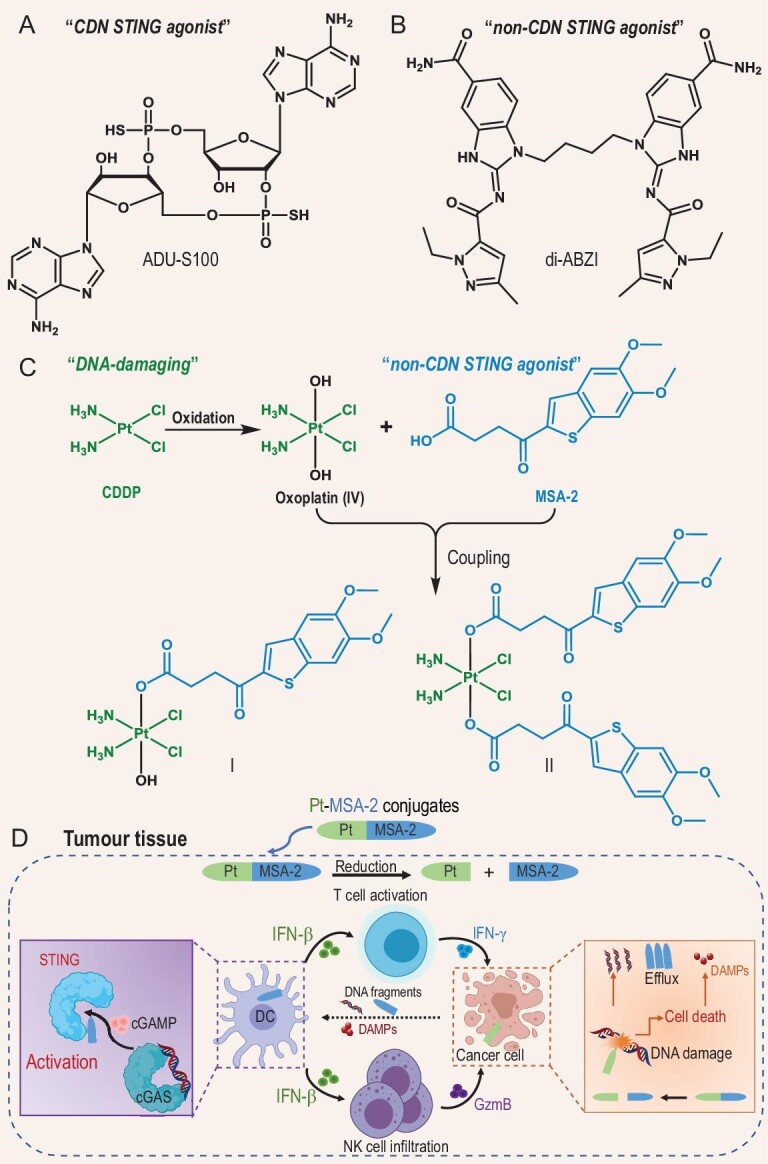
Design, synthesis and antitumor mechanism of Pt^IV^-MSA-2 conjugates **I** and **II**. Chemical structures of (A) the CDN-based STING agonist ADU-S100 and (B) the non-CDN-based STING agonist di-ABZI. (C) Schematic illustration of the construction of conjugates **I** and **II**. (D) Schematic illustration of the combinational effects on tumors of the Pt^IV^-MSA-2 conjugates. The Pt^IV^-MSA-2 conjugates are reduced to Pt^II^ species and MSA-2 in tumor tissues. The Pt^II^ species kill tumor cells, causing the release of DNA fragments and danger-associated molecular patterns (DAMPs) to activate STING in DCs, which is also amplified by free MSA-2. STING activation in DCs promotes IFN-β secretion to activate CD8^+^ T and NK cells, which release IFN-γ and GzmB for tumor killing. All these factors eventually amplify the antitumor therapeutic effects.

Cyclic guanosine monophosphate-adenosine monophosphate (cGAMP) synthase-stimulator of interferon genes (cGAS-STING), as a cytosolic DNA sensing pathway, participates in modulating fundamental innate immunity pathways [8]. Accumulating evidence suggests that the activation of the cGAS-STING pathway favors tumoricidal immunity [9]. The downstream interferons (IFNs) mediated by cGAS-STING can not only activate innate immunity but also prompt T-cell priming and tumor infiltration [10]. Accordingly, a growing number of studies have focused on the synthesis and screening of STING agonists [11]. The first generation of STING agonists were cyclic dinucleotide (CDN) analogues (Fig. 1A); however, their poor stability and cellular permeability and requirement for intratumoral delivery limited their application to a small set of tumors [12]. Recently, several non-CDN-based human STING agonists (e.g. di-ABZI and MSA-2) intended for systemic administration were reported to exhibit strong antitumor activity, showing great potential for the development of CITs targeting the STING pathway (Fig. 1B and C) [13–15]. On the other hand, endogenous cGAS-STING pathways are usually activated by double-stranded DNA (dsDNA) fragments released by diseased cells and recognized by cGAS, the cytosolic DNA sensor [16]. Accordingly, DNA-damaging chemotherapeutic drugs are usually thought to release dsDNA, which further activates the innate immune system through the cGAS-STING pathway [17]. It is obvious that combination therapy including these two regimens would likely achieve synergistic effects against tumors due to the initiation and amplification of cGAS-STING signaling. Indeed, a nanosystem concurrently delivering the chemotherapeutic agent SN38 and the STING agonist DMXAA has been reported to elicit a potent STING-mediated antitumor immune response in multiple murine models [18]. However, compared to combination therapy or single-pill combination therapy, ‘single-molecule multitarget’ drugs are favored in the clinic due to their unique advantages, including convenient administration, lower risk of drug interactions, more predictable pharmacokinetics and pharmacodynamics, and lower clinical trial cost, which may ultimately maximize drug efficacy and minimize side effects [19–22]. To our knowledge, almost no single-molecule multitarget drugs have been approved for small-molecule-based cancer immunotherapy. Herein, we developed two Pt^IV^-MSA2 conjugates (**I** and **II**) as single-molecule multitarget drugs by linking the newly identified non-CDN-based STING agonist MSA-2 to the Pt^IV^ cisplatin (CDDP) prodrug (Fig. 1C). CDDP is a widely used DNA-damaging metal anticancer drug in the clinic [23], and some reports have demonstrated that it can promote DNA fragment release in the TME to activate the cGAS-STING pathway [24]. CDDP and MSA-2 might be natural partners in a single-molecule multitarget drug design, as CDDP induces DNA fragment release during tumor cell killing [16], facilitating the remote activation of the cGAS-STING pathway in tumor-infiltrating immune cells, while MSA-2 could further enhance downstream intracellular STING signaling (Fig. 1D). Our results demonstrated that these conjugates could activate cancer-specific immune reactions and boost anticancer activity in a pancreatic cancer mouse model. As the Pt^IV^ prodrug strategy used here has been widely applied in the design of new Pt antitumor complexes [25–30], Pt^IV^-MSA-2 conjugates may have the potential to be tested in the clinic, possibly providing a new avenue for single-molecule multitarget drugs in cancer metalloimmunotherapy.

## RESULTS

### Synthesis and characterization of conjugates I and II

**Figure 2.**
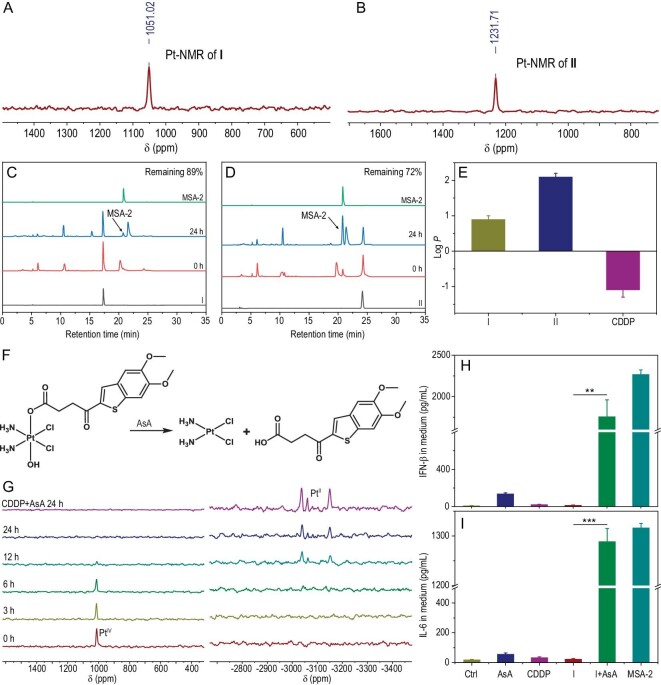
Characterization of Pt^IV^-MSA-2 conjugates **I** and **II**. ^195^Pt-NMR spectra of conjugates (A) **I** and (B) **II**. HPLC (325 nm) chromatograms of conjugates (C) **I** and (D) **II** in cell culture medium (containing 10% FBS, 37°C). (E) Partition coefficients of conjugates **I** and **II** and CDDP. (F) The reduction of conjugate **I** by AsA. (G) ^195^Pt-NMR spectra for the reaction of conjugate **I** with AsA at 37°C in the dark (left: low field; right: high field). (H) IFN-β and (I) IL-6 concentrations in supernatants of BMDCs incubated with different complexes or mixtures. I + AsA: conjugate **I** pretreated with AsA for 24 h. **P < 0.01, ***P < 0.001.

To create single-molecule multitarget drugs that can achieve STING activation and DNA damage, we synthesized Pt^IV^-MSA-2 conjugates **I** and **II** (Scheme S1). Their structures and purities were confirmed by ^1^H-, ^13^C- and ^195^Pt-nuclear magnetic resonance (NMR) and high-resolution electrospray ionization mass spectrometry (ESI-HRMS) (Fig. 2A and B, Figs S1–S6), which agreed with the preconceived structures of the conjugates. The proton resonances of the amine connected to the Pt^IV^ center ranged from 5.0 to 7.0 ppm, similar to those of other Pt^IV^ complexes [31]. The ^195^Pt-NMR spectra showed single resonance peaks at 1051.02 and 1231.71 ppm for conjugates **I** and **II**, respectively, which were also close to those of many other Pt^IV^ complexes [31]. The purities of these conjugates were further characterized by high-performance liquid chromatography (HPLC). The data showed that their purities were >95% (Fig. 2C and D), which satisfied the requirements for biological assessment. The stability of conjugates **I** and **II** in cell culture medium was further confirmed by HPLC (Fig. 2C and D). The octanol-water partition coefficients (logP_o/w_) of conjugates **I** and **II** were measured to be 0.9 and 2.1, respectively, implying that they have higher lipophilicity than CDDP (Fig. 2E).

It is widely accepted that Pt^IV^ complexes need to be reduced to Pt^II^ species by endogenous reductants such as ascorbic acids (AsA) to exert their antitumor activity [28]. Therefore, we studied the reduction process by monitoring the reaction between the conjugates and AsA using ^1^H-NMR and ^195^Pt-NMR. The peak at 8.16 ppm, corresponding to the thiophene ring of conjugates **I** and **II**, diminished and new peaks that were similar to the peak of MSA-2 appeared over time, indicating the release of the axial ligands under reducing conditions (Figs S7 and S8). The reduction of conjugates **I** and **II** finished at 24 and 120 h, respectively, indicating that the reduction rate of conjugate **I** was faster than that of conjugate **II**. Interestingly, the new group of peaks emerged between spectra of the original Pt complex and free MSA-2 after 12 h of incubation with AsA. To explain this, the ^1^H-NMR changes of the mixture of CDDP/MSA-2 with AsA were followed and similar peaks were found after 6 h of incubation, indicating that the peaks derived from a new species generated by CDDP/MSA-2 and AsA (Fig. S9). ^195^Pt-NMR data indicated similar trends in the reduction rates, and the peak for Pt^IV^ vanished and that for Pt^II^ appeared in the spectra over time (Fig. 2G and Fig. S10). The reduced Pt^II^ peak of conjugate **I** exhibited similarity to CDDP, indicating its reduction to CDDP (Fig. S11). In contrast, the reduction peak of conjugate **II** differed from that of CDDP, suggesting the presence of distinct Pt^II^ species. Furthermore, we wondered whether the species released by reduction could exert therapeutic effects, so we next studied the STING activation induced by these species. Activation of the STING protein triggers the release of the type I interferon IFN-β and the proinflammatory cytokine IL-6 [13], so we tested their levels in the supernatants of bone-marrow-derived dendritic cells (BMDCs), after treatment with the conjugates under different conditions for 18 h using enzyme-linked immunosorbent assay (ELISA). As shown in Fig. 2H and I, free MSA-2 induced robust IFN-β and IL-6 secretion, in line with a previous report [13]. In contrast, conjugate **I** alone triggered only minimal IFN-β and IL-6 secretion, which was significantly less than that induced by MSA-2. We speculated that the axial MSA-2 ligand might not be efficiently reduced for release in BMDCs; thus, we further measured the levels of these cytokines in BMDCs under treatment with conjugate **I** preincubated with the reducing agent AsA. The data showed that pretreatment of conjugate **I** with AsA resulted in significantly enhanced IFN-β and IL-6 secretion compared with no pretreatment. These results fully demonstrate that conjugate **I** is able to activate the STING pathway in a reduction-dependent manner, coincident with the properties of Pt^IV^ prodrugs, and may have fewer side effects from unwanted overactivation of the STING pathway in immune cells far away from the tumor.

### Pt-MSA-2 conjugate I kills cancer cells effectively via distinct mechanisms

The cytotoxicity of conjugates **I** and **II** towards a panel of cancer cell lines and a normal cell line was tested following 72 h of treatment; the cell lines included the human breast cancer cell line MCF-7, human ovarian cancer cell line Caov3, human pancreatic cancer (PC) cell lines PANC-1 and BxPC3, mouse PC cell line Pan02 and human normal kidney cell line HK-2. CDDP, MSA-2, oxoplatin and mixtures of CDDP and MSA-2 served as control complexes. Generally, conjugate **I** was more cytotoxic to these cell lines than CDDP or conjugate **II**, with half maximal inhibitory concentrations (IC_50_ values) in the submicromolar ranges (Table 1 and Table S1). The mixtures of CDDP and MSA-2 showed similar cytotoxicity to CDDP against these cells, suggesting that MSA-2 alone contributed minimally to the cytotoxicity. The IC_50_ value of MSA-2 was over 50 μM in these cancer cell lines. Notably, conjugate **I** exhibited striking cytotoxicity against all tested PC cell lines. The superior cytotoxicity of conjugate **I** against PC cells suggests its potential in the development of an anti-PC therapy, so we chose PC as a tumor model and conjugate **I** as a lead drug in the subsequent biological assays.

**Table 1.** IC_50_ values (μM) as inferred from 72 h MTT assays of Pt prodrugs and relevant compounds tested against cancer cell lines. 2MSA-2: MSA-2 is present at two times the equimolar amount of CDDP.

Compounds	HK-2	Caov3	PANC-1	BxPC3	Pan02
**I**	0.12 ± 0.01	0.7 ± 0.1	0.06 ± 0.02	0.02 ± 0.01	0.05 ± 0.01
**II**	3.6 ± 1.2	5.3 ± 0.4	6.9 ± 0.9	4.9 ± 0.8	8.4 ± 0.7
CDDP	8.2 ± 0.5	26.8 ± 2.2	11.1 ± 1.3	1.6 ± 0.1	8.3 ± 0.6
CDDP + MSA-2	9.9 ± 1.3	31.8 ± 1.3	11.0 ± 1.6	1.5 ± 0.1	10.0 ± 0.4
CDDP + 2MSA-2	11.4 ± 1.5	31.5 ± 1.1	11.3 ± 1.1	1.5 ± 0.1	10.9 ± 0.8
Oxoplatin	38.7 ± 4.1	>50	>50	>50	>50
MSA-2	> 50	>50	>50	>50	>50

**Figure 3.**
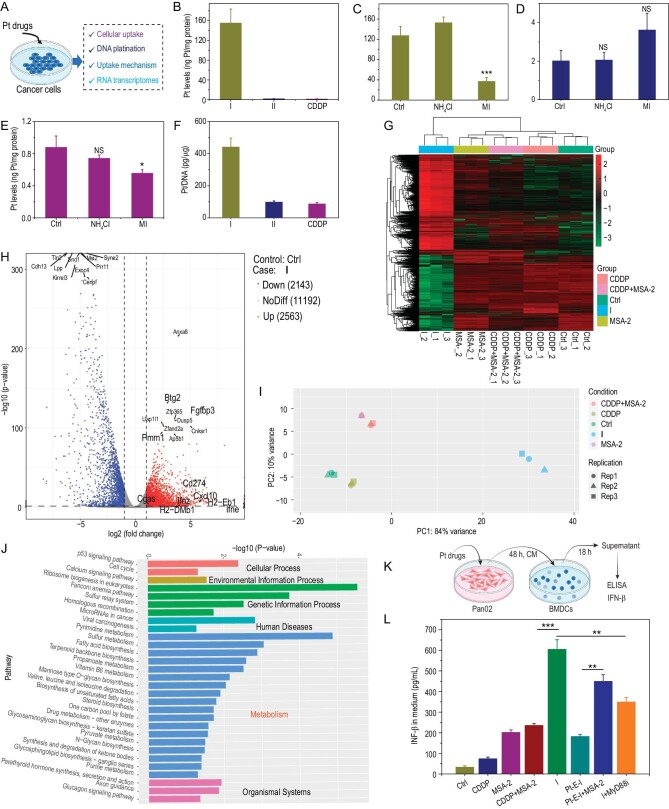
Pt-MSA-2 conjugate **I** kills cancer cells effectively via multiple mechanisms and induces cancer cell immunogenicity. (A) Schematic representing the experimental process for determining the mechanism of action in cancer cells. (B) Cellular uptake of Pt (ng Pt/mg protein) in PANC-1 cells after exposure to conjugate **I**, conjugate **II** or CDDP for 6 h. PANC-1 cells were incubated with (C) conjugate **I**, (D) conjugate **II** and (E) CDDP (5 μM) for 6 h. NH_4_Cl: cells preincubated with 50 mM NH_4_Cl; MI: cells preincubated with 50 mM 2-deoxy-D-glucose and 5 μΜ oligomycin. (F) Platination of cellular DNA in PANC-1 cells after incubation with these complexes. (G) Hierarchical clustering of differentially expressed genes between conjugate-**I**-treated Pan02 cells and all the other control-treated cells. (H) Volcano plot of upregulated (right) and downregulated (left) genes between the **I**-treated and untreated groups. Results with a *P*-value < 0.05 and |log_2_(fold change)| > 1 were considered significant. (I) PCA of all groups. (J) KEGG pathway enrichment analysis of differentially expressed genes in **I**-treated cells compared with untreated cells. (K) Schematic representing the experimental process for inducing IFN-β secretion by BMDCs. (L) IFN-β concentrations in the supernatants of BMDCs incubated with Pan02 cell CM. NS, not significant, *P < 0.1, **P < 0.01, ***P < 0.001 (versus the control group).

Subsequently, we comprehensively investigated the cytotoxic mechanism of **I**, including cellular uptake, DNA platination and transcriptome sequencing (Fig. 3A). The cellular Pt contents of PANC-1 cells were measured after incubation for 6 h by inductively coupled plasma-mass spectrometry (ICP-MS). Figure 3B shows that CDDP treatment for 6 h resulted in 1.7 ng Pt/mg protein in PANC-1 cells, and the numbers increased to 2.3 ng Pt/mg protein in conjugate-**II**-treated cells, which is in line with the lipophilicity of these drugs. In stark contrast, conjugate **I** accumulated much more efficiently. The level of Pt was 155.7 ng Pt/mg protein in PANC-1 cells after treatment with conjugate **I** for 6 h, showing 92- and 68-fold increases compared with CDDP and conjugate **II**, respectively. In view of the fact that conjugate **I** was less lipophilic than conjugate **II** (*vide supra*), the cellular uptake of conjugate **II** by simple passive diffusion should be more than that of conjugate **I**. However, the cellular accumulation of conjugate **I** was far higher than that of conjugate **II**, indicating that conjugate **I** might enter cells via a distinct pathway. We hypothesized that the cellular uptake of conjugate **I** is energy-dependent. PANC-1 cells were treated with 5 μΜ conjugate **I** for 6 h after preincubation with the metabolic inhibitors (MIs) 2-deoxy-D-glucose and oligomycin. The data showed that the cellular Pt levels significantly decreased in cells preincubated with the MIs compared to those without MI pretreatment (Fig. 3C), suggesting the significant role of energy-dependent transport in conjugate **I** cellular uptake. Energy-dependent transport includes active transport and endocytosis pathways. The possibility of uptake by endocytosis needed to be tested, as this process is involved in the uptake of many metal complexes [32]. PANC-1 cells were pretreated with the endocytosis inhibitor NH_4_Cl and then incubated with conjugate **I**. The intracellular Pt levels did not significantly change, indicating that active transport, not passive diffusion or endocytosis, is the major uptake pathway of conjugate **I**, which is distinct from the uptake routes of CDDP and conjugate **II** (Fig. 3D and E). This strong cellular uptake of conjugate **I** led to remarkable cytotoxicity. To our knowledge, one study has reported the development of an actively transported and highly potent Pt^IV^ complex (monochalcoplatin) [33]. Interestingly, that complex is also a monocarboxylated Pt^IV^ prodrug of CDDP, and the monocarboxylated Pt^IV^ prodrug of oxaliplatin with the same axial ligand showed no remarkable cellular accumulation. We thus speculated that the unique structures of CDDP and axial hydroxyl in conjugate **I** and monochalcoplatin may enable their energy-dependent active transport. Although the specific mechanisms of their active transport processes have not been explored, the discovery is still instructive for the design of new Pt^IV^ antitumor complexes.

The primary target of Pt anticancer drugs is nuclear DNA [34], so we assessed the ability of conjugates **I** and **II** to platinize nuclear DNA. Specifically, PANC-1 cells were treated with Pt complexes including CDDP and conjugates **I** and **II**, and then we extracted the genomic DNA by a DNA isolation reagent and finally measured the quantity of Pt in DNA by ICP-MS. Figure 3F shows that the platination of DNA by conjugate **I** was stronger than that by CDDP or conjugate **II**, which might be associated with the higher cellular accumulation of conjugate **I**. It is known that Pt^IV^ prodrugs are generally inert to DNA but can be activated to Pt^II^ equivalents by intracellular reducing agents for DNA binding [25]. As a result, the data for cellular DNA platination indirectly demonstrated that conjugates **I** and **II** were efficiently reduced to Pt^II^ species in cancer cells for DNA binding.

To better understand the mechanism of action of the highly cytostatic conjugate **I**, we compared the transcriptomes of Pan02 cells treated with conjugate **I**, CDDP, free MSA-2 or an equimolar mixture of CDDP and MSA-2. As shown in Fig. 3G, conjugate-**I**-treated Pan02 cells showed remarkably different transcriptional profiles compared to the cells in the control groups (including the non-, CDDP-, MSA-2- and ‘CDDP + MSA-2’-treated groups). Principal component analysis (PCA), a linear transformation that reduces high-dimensional data to two or three dimensions while retaining the features with the greatest contribution to each difference, clusters similar samples together, with closer distances indicating greater similarity between samples. PCA of all samples showed that the transcriptome phenotype of the conjugate **I** group was totally different from all controls, consistent with the results of the heat map (Fig. 3I). Then, the significant transcriptional differences between conjugate-**I**-treated and all control cells were analyzed (Fig. S12). As identified by comparisons of mRNA between the two groups using a volcano plot, 2563 and 2143 transcripts were significantly upregulated and downregulated, respectively (Fig. 3H). Innate immunity-related transcripts (*ifne, ifnz, Cxcl10*), the corresponding transcript for the DNA sensor cGAS (*Cgas*), major histocompatibility complex (MHC)-related transcripts (*H2-Eb1, H2-DMb1*), the clinically relevant transcript for the target PD-L1 (*Cd274*), and a fat and glucose metabolism associated transcript (*Fgfbp3*), as well as tumor suppressors (*Btg2, Pmm1*), were significantly overexpressed in conjugate-**I**-treated cells, suggesting that conjugate **I** is able to promote an immune reaction in cancer cells and affect metabolism-related pathways, which may facilitate anticancer immunity. Furthermore, we performed a bioinformatic analysis of all the differentially expressed mRNAs using Kyoto Encyclopedia of Genes and Genomes (KEGG) pathway screening (Figs S13–S15). As shown in Fig. 3J, conjugate **I** affected various metabolism-related pathways, such as pyrimidine, glutathione and sulphur metabolism, as well as the biosynthesis of steroids, fatty acids and N-glycans, but these pathways were not influenced in CDDP-treated cells (Fig. S13). These significant differences in gene expression could partly explain why the cytotoxicity of **I** is much higher than that of CDDP. Additionally, CDDP could activate the pathways associated with Pt drug resistance (Fig. S13), while the related pathways were not dramatically different in conjugate-**I**-treated cells, indicating that conjugate **I** might overcome Pt drug resistance, which is a key factor underlying failure of Pt chemotherapy in the clinic [35]. In addition, as the KEGG results indicated p53 pathway enrichment, we studied the cell death mode caused by conjugate **I**. The cytotoxicity of conjugate **I** was assessed by co-incubating conjugate **I** with various cell death inhibitors. As shown in Fig. S16, we observed a significant reversal of cell killing with administration of necroptosis and ferroptosis inhibitors, indicating that conjugate **I** may induce both necroptotic and ferroptotic cell death, which may potentiate antitumor immunity.

To further characterize the role of axial ligands in cytotoxicity, we employed a similar monosubstituted Pt^IV^ compound, Pt-E-I, which we had previously synthesized (Fig. S17A) as control complex. Firstly, we validated the cellular uptake mechanism of Pt-E-I and found it to also occur through energy-dependent active transport, similar to conjugate **I** (Fig. S17B). To eliminate the influence of Pt on cytotoxicity and the mechanism of action, we assessed the cellular uptake of both complexes at different concentrations and chose a treatment concentration where Pt uptake was comparable in the conjugate-**I-** and Pt-E-I-treated groups (Fig. S17C). We then performed cell viability assay and RNA transcriptome sequencing at this concentration (**I**, 5 μM; Pt-E-I, 20 μM), revealing that they exhibit comparable cytotoxicity, yet distinct transcriptional profiles (Figs S17D and E). These findings suggest that axial ligands may play a crucial role in cytotoxicity and the mechanism of action and provide valuable insights for future investigations into the mechanism of action of Pt^IV^ prodrugs.

### Pt-MSA-2 conjugate I enhances the immunogenicity of treated cancer cells

Cancer cells treated with DNA-damaging agents may release DNA fragments or other immunogenic molecules to activate the cGAS-STING pathway in neighboring antigen-presenting cells (APCs) [16]. Herein, we designed an experiment to evaluate the immunogenicity of these Pt prodrugs by culturing BMDCs with the supernatants of Pt drug-treated Pan02 cells. In brief, Pt drug-treated Pan02 cells caused the release of immunogenic substances to stimulate IFN-β secretion by BMDCs, which might be further enhanced through the release of the axial MSA-2 ligand in the conjugate-**I**-treated group. Specifically, these complexes were used to treat Pan02 cells for 48 h, and then the conditioned medium (CM) from each culture was transferred to BMDCs with an equal volume of fresh culture medium for another 18 h of incubation. The supernatants were subjected to ELISAs (Fig. 3K). The conjugate-**I**-treated Pan02 cell CM resulted in markedly increased IFN-β secretion compared to the untreated CM (Fig. 3L). The CDDP-treated Pan02 cell CM also resulted in slightly increased IFN-β secretion by BMDCs, but the level was far below that induced by conjugate **I**, which might be attributed to the stronger DNA platination and cytotoxicity induced by conjugate **I**. MSA-2-treated Pan02 cell CM also induced moderate IFN-β secretion, which could be attributed to the residual MSA-2 in the CM. Control complex Pt-E-I at 20 μM exhibited comparable cytotoxicity to conjugate **I** at 5 μM, but Pt-E-I-treated Pan02 cell CM induced lower IFN-β secretion compared to conjugate-**I**-treated Pan02 cell CM, indicating MSA-2 release in conjugate-**I**-treated Pan02 cells. As further evidence, ‘Pt-E-I + MSA-2’-treated Pan02 cell CM significantly elevated the levels of IFN-β, possibly because Pt-E-I-induced DNA fragmentation enhances the effects of MSA-2. ‘CDDP + MSA-2’-treated Pan02 cell CM induced a slightly higher level of IFN-β secretion compared to that of MSA-2, probably due to the low cancer cell killing of CDDP. Taken together, we hypothesize that IFN-β secretion induced by conjugate-**I**-treated Pan02 cell CM may be associated with the release of MSA-2 from dying cells. MyD88 serves as a crucial downstream molecule of DAMP sensors [36]. We pretreated BMDCs with a MyD88 inhibitor and observed a significant decrease in IFN-β secretion in the conjugate-**I**-treated Pan02 cell CM group, indicating the involvement of DAMP signaling pathways in IFN-β induction by conjugate **I**.

### *In vivo* antitumor activity of conjugate I and flow cytometric analysis of the tumor immune microenvironment

**Figure 4.**
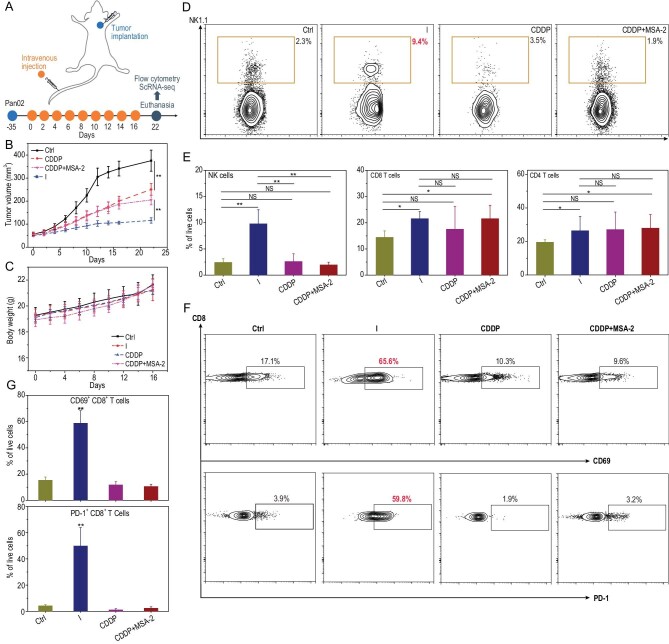
*In vivo* antitumor activity and flow cytometric analysis of the tumor immune microenvironment. (A) Schematic diagram of the *in vivo* antitumor experimental timeline. (B) Tumor growth and (C) body weight are shown (*n* > 5, data are shown as the mean ± SD; **P < 0.01). (D) Percentages of NK cell subpopulations in tumor tissue. (E) Statistical analysis of the proportions of NK cells (left), CD8^+^ T cells (middle) and CD4^+^ T cells in each group. *n* = 3, *P < 0.1, **P < 0.01 and NS = not significant (versus the control group). (F, G) Flow cytometric analysis of CD8^+^ T cells in Pan02 tumor tissues. *n* = 3, **P < 0.01 and NS = not significant (versus the control group).

To comprehensively evaluate the immunotherapeutic properties of **I**, we investigated immunocompetent C57BL/6 mice bearing Pan02 tumors as an animal model. The therapeutic efficacy of conjugate **I** and controls composed of complexes or mixtures were assessed following the therapeutic schedule shown in Fig. 4A. As shown in Fig. 4B, after treatment every 2 days for 16 days, conjugate **I** effectively suppressed tumor volume to 106.3 ± 6.2 mm^3^. However, the average tumor volumes for the control, CDDP-treated and CDDP + MSA-2-treated (mixture of CDDP with MSA-2 at a molar ratio of 1:1) groups were 341.2 ± 32.0, 200.2 ± 20.6 and 187.2 ± 21.8 mm^3^, respectively, which were obviously larger than that of the conjugate-**I**-treated group. The therapeutic efficacy of CITs is generally hysteretic; therefore, we continued to monitor tumor volume for another 6 days. The data showed that tumor growth was substantially suppressed following the cessation of conjugate **I** treatment, implying the induction of long-term anticancer immunity. No obvious changes in body weight were observed in C57BL/6 mice during the treatment period (Fig. 4C). Histological images of major organs, including the heart, liver, spleen, lungs and kidneys after treatment were also evaluated. The results showed that conjugate **I** induced only mild vacuolar degeneration in the liver and aggregates containing only a few inflammatory cells in the lungs, while substantial blood cell stasis in the liver blood sinuses and thickening of the alveolar wall as well as large infiltrates of inflammatory cells in the trachea and bronchus were observed in the CDDP-treated group (Fig. S19). These results demonstrate that conjugate **I** has high antitumor activity and low systemic toxicity in immunocompetent mice.

Since Pan02 tumors are poorly immunogenic and resistant to checkpoint blockade [37,38], we further investigated the immune activation profiles of conjugate-**I**-treated Pan02 tumors to characterize the immunological mechanism. As shown in Fig. 4D, intravenous delivery of conjugate **I** significantly increased the frequency of natural killer (NK) cells among tumor tissue cells, while CDDP and ‘CDDP + MSA-2’ treatment did not produce similar effects. NK cells are cytotoxic innate immune cells that are able to spontaneously detect and lyse stressed or malignant cells [39]. Increasing data have demonstrated the important role of intratumoral NK cells in driving the response to immunotherapy [39]. The increased infiltration of NK cells in conjugate-**I**-treated tumors thus suggested that conjugate **I** could promote an antitumor innate immune response, which agrees with the activation of the STING pathway. In fact, some studies have reported that NK cells are dominant in the tumour-cell-derived cyclic guanosine monophosphate-adenosine monophosphate (cGAMP)-stimulated STING pathway [40] and mediate the elimination of CD8^+^ T-cell-resistant tumors in response to STING agonists [41].

In contrast, the infiltration of CD8^+^ and CD4^+^ T cells was slightly increased in the conjugate-**I**-treated group; however, the infiltration was comparable to that in the CDDP and ‘CDDP + MSA-2’ groups (Fig. 4E and Fig. S21). Interestingly, the percentages of activated CD8^+^ T cells (CD69^+^CD8^+^, PD-1^+^CD8^+^) were markedly increased in the conjugate-**I**-treated group (Fig. 4F and G), in line with previous reports that the STING pathway can activate cytotoxic CD8^+^ T cells [13] and suggesting that conjugate **I** could not only activate innate immune cells but also induce a robust adaptive immune response.

Taken together, these results thus showed that conjugate **I** could switch the immunosuppressive status to an immune active status in an immunotherapy-resistant pancreatic tumor model, which relied on sustained activation of the cGAS-STING pathway and subsequent full activation of both innate and adaptive immune processes.

### Single-cell RNA-sequencing analysis of conjugate-I-treated tumors

**Figure 5.**
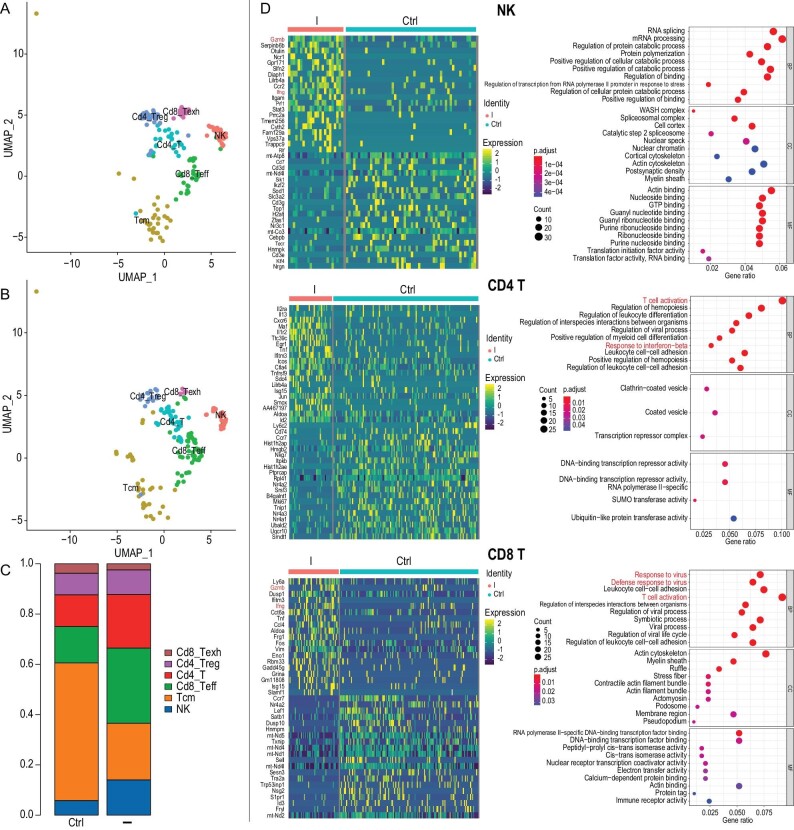
scRNA-seq showed the distinct immune cell activation signature of conjugate-**I**-treated Pan02 tumors. (A) Unsupervised clustering of lymphocytes derived from tumors excised from untreated mice and pooled. (B) The clusters in (A) colored by group (control versus conjugate-**I**-treated group). (C) Frequencies of the indicated cell clusters in the untreated and treated groups. (D) Heat map showing the relative expression of transcripts in NK, CD4^+^ and CD8^+^ T cells from the control and conjugate-**I**-treated groups (left); GO analysis of upregulated transcripts in NK, CD4^+^ and CD8^+^ T cells from the conjugate-**I**-treated group compared with those from the control groups (right).

To achieve a deep understanding of the full picture of immune landscapes in tumors triggered by conjugate **I** treatment, we excised tumors for single-cell RNA sequencing (scRNA-seq) analysis at the treatment endpoint and then analyzed the scRNA-seq data to reveal the gene signature of each cell inside the tumors. Unsupervised clustering analysis of the cells revealed populations that we identified, based on lineage and phenotypic marker expression, as cancer cells, T cells, myeloid cells and B cells (Fig. S22). The results showed that the percentage of tumor cells was decreased (Fig. S23), implying that conjugate **I** killed tumor cells effectively. Furthermore, lymphocyte clustering analysis revealed populations that we identified, based on lineage and phenotypic marker expression, as NK cells, central memory T cells, CD8 effector T cells, CD4 T cells, CD4 regulatory T cells and CD8 exhausted T cells (Fig. S24). The data are shown in Fig. 5. Conjugate **I** increased the frequencies of CD8 effector T (Cd8_Teff), CD4 T (Cd4_T) and NK cells, consistent with the flow cytometry data. Next, we compared the differentially expressed genes in T cells and NK cells between the control and conjugate-**I**-treated groups. As shown in Fig. 5D and Fig. S25, conjugate **I** treatment induced effector gene expression signatures of T cells and NK cells compared to no treatment. For example, genes encoding immune-cell-activating cytokines, such as *ifng*, and genes encoding certain canonical cytolytic molecules, such as *Gzmb*, were increased in CD8^+^ T cells and NK cells after conjugate **I** treatment. These results indicated that conjugate **I** could activate T and NK cell immunity, in agreement with the flow cytometry data.

Further gene ontology (GO) and KEGG analyses revealed that the T-cell activation-, response to IFN-β- and innate immune-related antivirus pathways were enriched in CD4^+^ and CD8^+^ T cells from the conjugate-**I**-treated group (Fig. 5D). In addition, pathways associated with NK-cell-mediated cytotoxicity in NK cells and innate immune-related pathways in dendritic cells (DCs) were also enriched (Fig. 5D, Figs S26 and S27). These general pathway enrichment analyses further indicated the full activation of cytotoxic immune profiles in conjugate-**I**-treated Pan02 tumors.

Last but not least, myeloid cell clustering analysis revealed populations that we identified, based on lineage and phenotypic marker expression, as neutrophils, DCs, myeloid-derived suppressor cells (MDSCs) and macrophages (Figs S28 and S29). Furthermore, we performed a comparative analysis of differentially expressed genes and GO enrichment analysis in myeloid cells between the conjugate-**I**-treated group and the untreated group. The results revealed enrichment of pathways associated with antiviral responses, responses to IFN-β and responses to IFN-γ, suggesting global activation of innate and adaptive immunity (Figs S30–S33).

## DISCUSSION

CITs have become promising cancer therapeutics, but their efficacy is constrained due to the immunosuppressive TME. Recently, activation of the cGAS-STING pathway has been considered to be an effective way to remodel the TME owing to the full activation of innate and adaptive immunity. Many cGAS-STING-activating compounds are being developed into CITs, but most of them are direct STING agonists; small molecules simultaneously stimulating the upstream cGAS and STING protein itself are rarely seen, and may be more effective than STING agonism alone and represent a new direction in the design of STING agonists. In this study, two Pt^IV^-MSA-2 conjugates (**I** and **II**) were designed by combining the DNA-damaging agent CDDP and the STING agonist MSA-2 into one molecule. CDDP induces DNA fragment release during tumor cell killing, which is recognized by cGAS in tumor-infiltrating immune cells to facilitate downstream STING activation, while MSA-2 can further enhance this signaling. Interestingly, conjugate **I** was highly cytotoxic to various cancer cell lines and, more importantly, showed significant antitumor activity in immunocompetent C57BL/6 mice bearing Pan02 tumors. Notably, the Pan02 tumor model used here is reported to be insensitive to checkpoint inhibitors [37,38]. Mechanistically, conjugate **I** could enter cancer cells via energy-mediated active transport and was later reduced to CDDP for DNA binding. RNA-sequencing analysis indicated that conjugate **I** but not CDDP significantly upregulated the expression of genes related to the innate immunity process and metabolism. For example, the pyrimidine metabolism pathway was significantly affected by conjugate **I**, which is rarely reported for other Pt-based drugs. Interestingly, the pyrimidine antimetabolite gemcitabine is usually used in combination with CDDP in the clinic to maximize efficacy. On the other hand, conjugate **I** could also activate the STING pathway in DCs in a reduction-dependent manner or by prompting cancer cells to release immunogenic substances. Analysis of treated tumor tissue via flow cytometry and single-cell RNA-sequencing demonstrated that conjugate **I** could enhance the infiltration of NK cells into tumors and promote the activation of T cells, NK cells and DCs in tumors, further triggering a strong immune response to inhibit tumor growth. These results proved that incorporating a DNA-damaging agent and STING agonist into one molecule can amplify STING activation and thus induce full activation of innate and adaptive immunity, having promise as a future CIT strategy.

PD-1 and PD-L1 are mainly expressed on T cells and DCs interacting with tumor-infiltrating lymphocytes (TILs), respectively, and their interaction attenuates T-cell activation [42]. Conjugate **I** induced an increased proportion of PD-1^+^CD8^+^ T cells and the activation of DCs in TILs (Fig. 4G). These results provide insight into the immune-related molecular signatures of conjugate**-I**-based therapy and suggest possible combination regimens, such as those including combination with anti-PD-1 and anti-PD-L1 therapy.

In summary, this study provides a novel single-molecule multitarget drug for CIT that was generated by combining the DNA-damaging agent CDDP and the STING agonist MSA-2. As discovered by a recent milestone study of drug combinations, synergy between drugs is rare and highly context-dependent [43]. Thus, our work demonstrated that single-molecule-based drug combinations might represent an effective approach in the future. The easy administration and dosage of single-molecule drug combinations also make them more favorable options in the clinic. Finally, it is worth mentioning that during the review process of our manuscript, a recent work showed that chronic activation of cGAS-STING is associated with cancer cell metastasis [44], and through this conjugation with CDDP, the STING agonist MSA-2 can enter cancer cells in large quantities, potentially disrupting the chronic activation of this pathway. We look forward to further validation of this mechanism of action in clinically relevant models.

## MATERIALS AND METHODS

All chemicals were commercially available and used without further purification. CDDP was purchased from Shandong Boyuan Pharmaceutical Co., Ltd. MSA-2 was purchased from Tianjing Heowns Biochemical Technology Co., Ltd. O-(Benzotriazol-1-yl)-*N,N,N*',*N*'-tetramethyluronium tetrafluoroborate (TBTU), Et_3_N and 3-(4,5-dimethyl-2-thiazolyl)-2,5-diphenyl tetrazolium bromide (MTT) were purchased from Energy Chemical. AsA, hydrogen peroxide (30% H_2_O_2_ aq), hydrochloric acid and nitric acid were purchased from Shanghai Aladdin Biochemical Technology Co., Ltd. Cis-diamminedichloro-trans-dihydroxyplatinum (oxoplatin) was prepared according to the literature [45].

Roswell Park Memorial Institute-1640 (RPMI-1640) medium and Dulbecco's modified Eagle medium (DMEM) were purchased from Gibco. Fetal bovine serum (FBS) (HyClone) was purchased from Thermo Scientific. A genomic DNA kit was acquired from TIANGEN Biotech Co., Ltd. All antibodies used for flow cytometric analysis were purchased from BioLegend. All ELISA kits were obtained from 4A Biotech Co., Ltd. The MyD88 inhibitor TJ-M2010-5 was purchased from MedChemExpress.

The human breast carcinoma cell line MCF-7, ovarian carcinoma cell line Caov3, PC cell line PANC-1, human normal kidney cell line HK-2 and mouse PC cell line Pan02 were purchased from American Type Culture Collection (ATCC). C57BL/6 mice were purchased from the Model Animal Research Center of Nanjing University. scRNA-seq associated agents were obtained from 10X Genomics Chromium Controller. ^1^H-NMR, ^13^C-NMR and ^195^Pt-NMR results were recorded at 296.5 K in d_6_-DMSO on a Bruker AVANCE III HD 400 MHz with trimethylsilyl (TMS) as the internal reference. High-resolution mass spectrometry (HR-MS) data were collected with a Thermo Scientific Q Exactive Orbitrap. HPLC results were recorded on a Thermo Scientific UltiMate 3000 with an Ace Excel 5 C18 column (250 mm × 4.6 mm, Guangzhou FLM Scientific Instrument Co., Ltd.). MTT assay data were acquired with a Hangzhou Allsheng AMR-100 microplate reader. Pt content was determined on an inductively coupled plasma mass spectrometer using a standard Plasma-Quad II instrument (VG Elemental, Thermo OptekCorp.). Flow cytometry was performed in an Agilent NovoCyte Quanteon.

## Supplementary Material

nwae020_Supplemental_File
