# Supplementary material for: Combining cisplatin and a STING agonist into one molecule for metalloimmunotherapy of cancer
Source: Natl Sci Rev. 2024 Jan 17;11(1):nwae020. doi: 10.1093/nsr/nwae020 (PMC10852989; doi:10.1093/nsr/nwae020)
Supplement: nwae020_Supplemental_File [file nwae020_supplemental_file.docx]

Supplementary Materials for

**Combining cisplatin and a STING agonist into one molecule for metalloimmunotherapy of cancer**

Shuren Zhang, Dongfan Song, Wenhao Yu, Ji Li, Xiaoyu Wang, Yachao Li, Zihan Zhao, Qi Xue, Jing Zhao, Jie P. Li*, and Zijian Guo*

*Corresponding author. E-mail: jieli@nju.edu.cn (J. P. L.); zguo@nju.edu.cn (Z. J. G)

**Synthesis of I.** Oxoplatin (100 mg, 0.3 mmol) was stirred in dry DMF (5 mL). MSA-2 (53 mg, 0.181 mmol), TBTU (58 mg, 0.181 mmol) and TEA (18 mg, 0.180 mmol) were mixed in dry DMF (5 mL) and then slowly added to the reaction. The mixture was stirred at room temperature for 24 h in the dark. The solution obtained was centrifuged to remove the precipitate. The supernatant was concentrated to about 2 mL and then was added to a mixture of ice-cold H_2_O and EtOH (20 mL, 3:1) to give a grey precipitate. The precipitate was washed twice with cold EtOH and diethyl ether and then dried under vacuum. Conjugate **I** was obtained as a solid in a yield of 68.2%. ^1^H-NMR peaks (400 MHz, DMSO-*d*_6_): δ 8.15 (s, 1H), 7.59 (s, 1H), 7.49 (s, 1H), 6.17–5.70 (m, 6H), 3.86 (s, 3H), 3.83 (s, 3H), 3.18 (t, *J* = 6.8 Hz, 2H), 2.61 (t, *J* = 6.8 Hz, 2H), and 0.99 (br, 1H). ^13^C-NMR peaks (101 MHz, DMSO-*d*_6_): δ 193.04, 179.67, 150.61, 148.38, 140.86, 135.50, 132.44, 129.98, 106.52, 104.15, 55.74, 55.50, 34.25, and 30.43. ^195^Pt-NMR peak (86 MHz, DMSO-*d*_6_): δ 1051.02. ESI-HRMS result: calculated, 631.9959 {[C_14_H_20_Cl_2_N_2_O_6_PtS+Na]^+^}; found, 631.9951 {[M+Na]^+^}.

**Synthesis of II.** Oxoplatin (100 mg, 0.3 mmol) was stirred in dry DMF (10 mL) with MSA-2 (265 mg, 0.9 mmol), TBTU (290 mg, 0.9 mmol) and TEA (91 mg, 0.9 mmol) at room temperature for 60 h in the dark. The solution obtained was centrifuged to remove the precipitate. The supernatant was concentrated to about 2 mL and then added to a mixture of H_2_O and EtOH (20 mL, 1:1) to give a grey precipitate. The precipitate was washed twice with MeOH and diethyl ether and then dried under vacuum. Conjugate **II** was obtained as solid in a yield of 65.2%. ^1^H-NMR peaks (400 MHz, DMSO-*d*_6_): δ 8.16 (s, 2H), 7.59 (s, 2H), 7.49 (s, 2H), 6.51 (br, 6H), 3.86 (s, 6H), 3.83 (s, 6H), 3.18 (t, *J* = 6.7 Hz, 4H), and 2.69 (t, *J* = 6.7 Hz, 4H). ^13^C-NMR (101 MHz, DMSO-*d*_6_): δ 192.75, 179.68, 150.75, 148.51, 140.81, 135.64, 132.53, 130.15, 106.61, 104.26, 55.85, 55.61, 33.98, and 29.63. ^195^Pt NMR (86 MHz, DMSO-*d*_6_): δ 1231.71. ESI-HRMS result: calculated, 908.0415 {[C_28_H_32_Cl_2_N_2_O_10_PtS_2_+Na]^+^}; found, 908.0401 {[M+Na]^+^}.

**Stability of I and II.** Conjugates **I** and **II** were dissolved in DMEM containing 10% FBS or 0.5% DMSO at 10 μM, and stored at 37 °C for 24 h. Reversed phase (RP) HPLC was used to monitor the residual prodrugs at 325 nm.

**Reduction of I and II.** Samples were prepared by reacting **I** or **II** (20 mM) with 8 equiv. AsA in 5:1 d_6_-DMSO:D_2_O solutions. The time course of the reaction was monitored by ^1^H-NMR and ^195^Pt-NMR after incubation of the samples were incubated at 37 °C for 0, 3, 6, 12, 24, 36, 48, 60, 72, 84, 96, or 120 h in the dark.

**Lipid-water partition coefficient.** The shake-flask method was used to measure the partition coefficients of CDDP, **I** and **II**. Briefly, a mixture containing equal volumes of n-octanol and phosphate buffer (10 mM, pH 7.4) was shaken on a mechanical shaker at room temperature for 24 h. Complexes (10 mM) in phosphate buffer (presaturated with n-octanol) were mixed with an equal volume of n-octanol (presaturated with phosphate buffer), and the mixture was shaken for 24 h at room temperature. Centrifugation was carried out at 2500 rpm for 30 min to separate the two phases. The aqueous layer was carefully separated, and the Pt content in the initial and final aqueous phases was analyzed by inductively coupled plasma mass spectrometry (ICP-MS). The log *P_O/W_* was measured using the following equation: log *P_O/W_* = log [(Pt_initial_ ‒ Pt_final_)/Pt_final_].

**Cytotoxicity.** Tumor cells were cultured overnight after inoculation in RPMI-1640 medium or DMEM supplemented with 10% FBS. All cultures were maintained in an incubator in a highly humidified atmosphere of 95% air with 5% CO_2_ at 37 °C. Growth inhibition was measured with an MTT assay. Briefly, 2800–7000 cells per well in culture medium (100 μL) were plated in 96-well plates (Corning). The cells were treated in triplicate with different concentrations of complexes at 37 ℃ for 72 h. Stock solutions of CDDP and oxoplatin were prepared in PBS, while stock solutions of conjugates **I** and **II** and the ligand MSA-2 were prepared in DMSO. The stock solutions were diluted in complete medium (DMSO < 0.5%). An aliquot of MTT solution (40 μL, 2.5 mg·mL^–1^) in PBS buffer was added to each well and incubated for 4 h. The supernatant was removed and DMSO (150 μL) was added to solubilize the formazan crystals. The amount of formazan was determined using a microplate reader at 570 nm after the plates were shaken for 30 min. The optical density (OD) was used to calculate the percentage of viable cells relative to untreated control values. Background readings for MTT incubated in cell-free medium were subtracted from each value before calculation. The half maximal inhibitory concentration (IC_50_) values of the complexes were obtained from fitted inhibition curves at 72 h. The mean IC_50_ values were calculated using data from three replicates.

For Pan02 cells, the cell viability in the presence of different cell death inhibitors was also determined. Therefore, Pan02 cells were respectively pre-incubated with z-VAD-fmk (50 μM), 3-Methyladenine (100 μM), ferrostatin (50 μM), necrostatin-1 (50 μM) 1 h, and then co-incubated **I** (0.2 μM) for 72 h. The cell viability was then determined.

**Cellular uptake.** All incubations were carried out in the dark. After the designated incubation period, the medium was removed, and the cells were harvested with trypsin and rinsed twice with PBS (1×, 20 mM, pH 7.4), followed by digestion and ICP-MS.

Cancer cells were seeded in a 6-well plate at a density of 5×10^5^ cells per well. After incubation for 24 h, the cells were treated with each complex for 6 h.

Cancer cells preincubated with metabolic inhibitor (MIs) were also evaluated. These cells were preincubated with 2-deoxy-D-glucose and oligomycin (50 mM and 5 μM, respectively, 1 h) or NH_4_Cl (50 mM, 1 h) at 37 ℃ followed by incubation with DMEM containing CDDP, **I** or **II** at 37 ℃ for 6 h.

**Digestion.** Cell pellets were collected by centrifugation and then digested with nitric acid (100 µL) for 2 h at 95 °C, followed by the addition of H_2_O_2_ (50 µL) at 95 °C for 0.5 h. HCl (100 µL) was added and kept at 95 °C until the total volume was less than 20 μL. Water was then added to dilute the solution to 1 mL, and the final Pt content was determined by ICP-MS.

**DNA platination.** Cancer cells were seeded in 6-well plates at a density of 5×10^5^ cells per well. After incubation at 37 °C for 24 h, the cells were treated with each complex for 6 h. The attached cells were harvested with trypsin and washed twice with PBS (4 °C). The cell pellets were lysed with the TIANamp Genomic DNA Kit. The DNA concentration was determined using NanoDrop spectrophotometer, and then the isolated DNA was digested with the same method mentioned above. The final Pt content was determined by ICP-MS.

**Bulk mRNA sequencing.** Pan02 cells were seeded in 6-well plates at a density of 1×10^6^ cells per well. After incubation at 37 °C for 24 h, the cells were treated with each complex at 5 μM for 20 h. The attached cells were washed twice with PBS (4 °C), harvested with TRIzol, cryopreserved at -80 °C, and then sent to Personalgene Technology Co., Ltd. for bulk mRNA sequencing. Briefly, we first used HTSeq (0.11.1)(1) statistics to compare read count values of each gene as the original expression of the gene and then used FPKM to standardize the expression. Then, we used DESeq (1.39.0)(2) to analyze the genes with differential expression identified under the following screening conditions: expression difference multiple |log_2_FoldChange| > 1 and significant P-value < 0.05. At the same time, we used the R language Pheatmap (1.0.12) software package to perform bidirectional clustering analysis of all the differential expressed genes in the samples. We obtained a heatmap showing the expression levels of the same gene in different samples and the expression patterns of different genes in the same sample with the Euclidean method to calculate the distance and the complete linkage method to perform clustering. Next, we used the top Gene Ontology (GO) (2.40.0) to map all the genes to terms in the GO database and calculated the numbers of differentially enriched genes in each term. Using top GO to perform GO enrichment analysis on the differential genes, calculate P-value by hypergeometric distribution method, and find the GO term with significantly enriched differential genes to determine the main biological functions performed by differential genes. The purpose of GO enrichment analysis was to identify GO functional terms with significant enrichment of differentially expressed genes, thus revealing the possible functions of the differentially expressed genes in the samples. Additionally, we counted the number of differentially expressed genes at different levels of Kyoto Encyclopedia of Genes and Genomes (KEGG) pathway analysis and then determined the metabolic pathways and signaling pathways in which differentially expressed genes mainly participated. ClusterProfiler (3.4.4) software was used to carry out the enrichment analysis of the KEGG pathway of differential genes, focusing on the significant enrichment pathway with P-value <0.05.

**ELISA.** Bone marrow-derived dendritic cells (BMDCs) were prepared according to the following method. Briefly, femurs and tibias were removed from 4-6 week-old C57BL/6 mice, and the bone marrow cells were isolated. The bone marrow cells were incubated in T cell medium [RPMI-1640 medium (with GlutaMAX), supplemented with 10% FBS, 1% HEPES, 1% NEAA, 1% sodium pyruvate, and 0.1% βME] containing 20 ng/mL GM-CSF for 6 days after erythrocyte lysis, during which time half of the medium was replaced with fresh T cell medium once and the cytokines were supplemented. Then, the BMDCs were harvested on day 7 for further study.

Pan02 tumor cells were treated with various drugs at a concentration of 5 μM for 48 h, and then the drug-pretreated tumor cell supernatant was obtained by centrifugation at a speed of 12000 rpm/min for 5 min and used as conditioned media (CM) subsequently.

BMDCs were cultured with 50% CM plus 50% fresh medium for 18 h. In addition, BMDCs were cultured with fresh medium containing different complexes or mixtures (15 μM CDDP and **I**, a mixture of **I** and 8 equiv. AsA, and equal AsA). Then, all the BMDC supernatant was collected and centrifuged for subsequent cytokine analysis using ELISA kits, which were performed according to the manufacturer’s instructions.

Pan02 tumor cells were treated with various drugs at a concentration of 5 μM for 24 h, and then the drug-pretreated tumor cell supernatant was obtained by centrifugation at a speed of 12000 rpm/min for 5 min and used for subsequent cytokine analysis (Cxcl10) using ELISA kits, which were performed according to the manufacturer’s instructions.

***In vivo* antitumor activity.** Female C57BL/6 mice (6–8 weeks old) were subcutaneously implanted with pan02 cells (2 × 10^6^) in the right flank. Once the tumor volume reached approximately 60 mm^3^, the mice were assigned to 4 groups at random (n = 5–8) and received treatment with PBS, CDDP (10.0 μmol/kg), **I** (10.0 μmol/kg), or a mixture of CDDP (10.0 μmol/kg) and MSA-2 (10.0 μmol/kg). The mice received 9 treatments. Changes in body weight and tumor volume were recorded. All the experimental procedures utilizing mice were performed in accordance with the Guidelines for Care and Use of Laboratory Animals of Nanjing University and experiments were approved by the Institutional Animal Care and Use Committee of Nanjing University (IACUC-2101001, SL-019-00).

**Hematoxylin and eosin staining.** Tissue samples were subjected to histopathological examination using hematoxylin and eosin (H&E) staining. Mouse organs (heart, liver, spleen, lungs and kidneys) were collected in 4% paraformaldehyde for proper fixation and then embedded in paraffin using a tissue embedding machine. Sections were prepared in an orderly manner by dewaxing, dehydration, and H&E staining. The tissue morphology was observed under a fluorescence microscope (Olympus BX41, Japan).

**Flow cytometric analysis.** To analyze the levels of immune cells in the tumor microenvironment, mice were sacrificed six days after the final treatment. The obtained tumors were cut into pieces and digested in T cell medium. Single-cell suspensions were obtained after filtration through nylon mesh and incubated with various antibodies. FACS tests were carried out on a flow cytometer, and the results were analyzed with NovaExpress. The following monoclonal antibodies (mAbs) were used for flow cytometry: anti-CD3-APC (17A2), anti-CD4-AF700 (RM4-5), anti-CD8-PE (53-6.7), anti-NK1.1-PE/Cy7 (PK136), anti-PD-1-FITC (29F. 1A12), anti-CD69-PE/Cy5 (H1.2F3).

**ScRNA-seq.** Tumors were excised from individual mice after treatment using a dissecting microscope and pooled. The tumors were cryopreserved in FBS + 10% DMSO. Samples were subsequently thawed, washed, sorted to isolate living cells and then performed on the 10X Genomics 5’ Chromium v2.0 platform according to the manufacturer’s instructions. Libraries were generated per the manufacturer’s protocol and sequenced on an Illumina HiSeq 2500 with the “rapid run” mode according to the standard 10X Genomics protocol. Data analysis was conducted by by Singleron Biotech. Co. Ltd. using Cellranger and R.

**Cell type annotation.** The cell type identity of each cluster was determined from the expression of canonical markers found in the DEGs using SynEcoSys database. Heatmaps displaying the expression of markers used to identify each cell type were generated by Seurat v3.12 DoHeatmap.

**Pathway enrichment analysis.** To investigate the potential functions of immune cells, GO and KEGG analyses were performed with the “clusterProfiler” R package version (version 4.0.2, <https://bioconductor.org/packages/release/bioc/html/clusterProfiler.html>). Pathways with a p_adj value less than 0.05 were considered significantly enriched.(3-5)

**Statistical analysis.** Statistical analyses were performed using GraphPad Prism software (version 7.0) and Excel. Comparisons among groups were analyzed using two-way ANOVA followed by Tukey’s multiple comparisons test, and comparisons of multiple samples with one group were analyzed using two tailed t test or one-way ANOVA followed by Tukey’s multiple comparisons test. In all figures with error bars, data are presented as the mean ± SD.

**Scheme S1.** The synthesis of Pt^IV^-MSA-2 conjugates **I** and **II**.


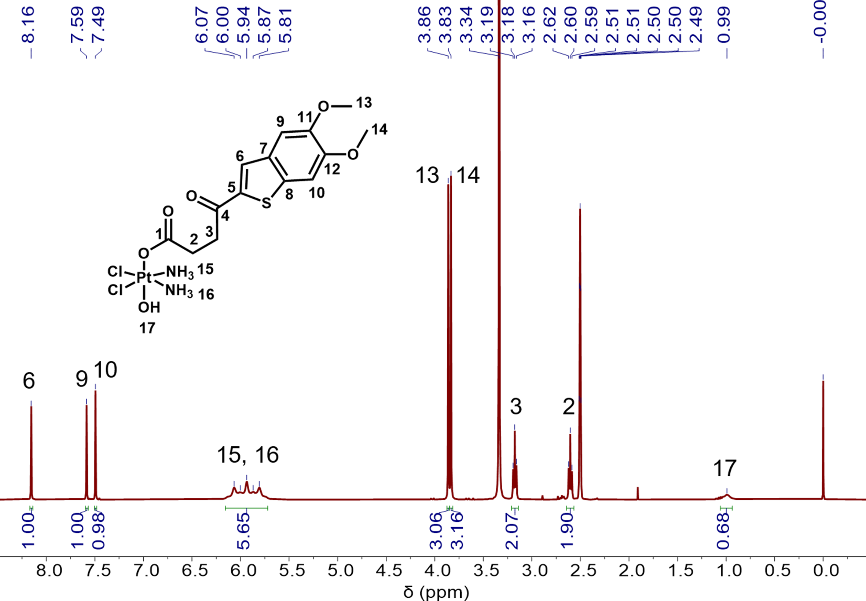


**Fig. S1.** The ^1^H-NMR spectrum of **I**.


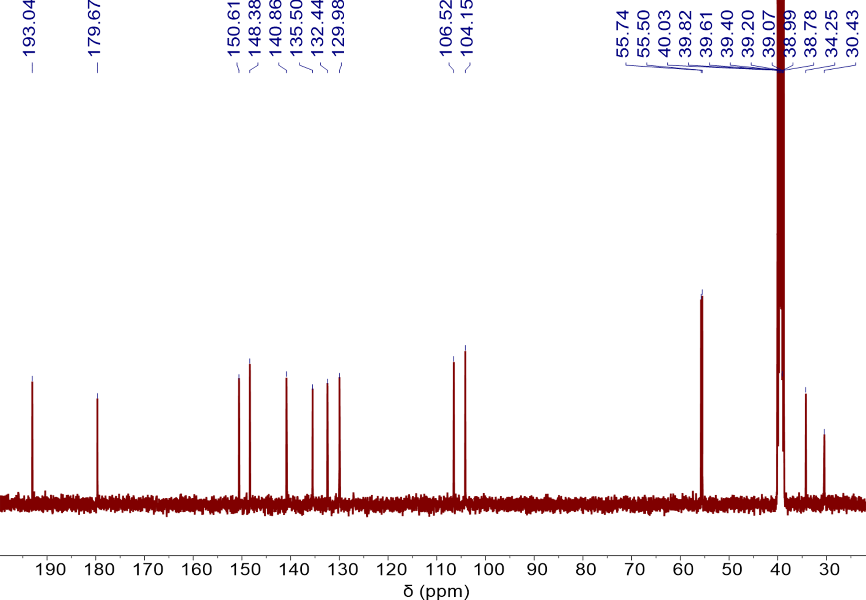


**Fig.S2.** The ^13^C{^1^H}-NMR spectrum of **I**.


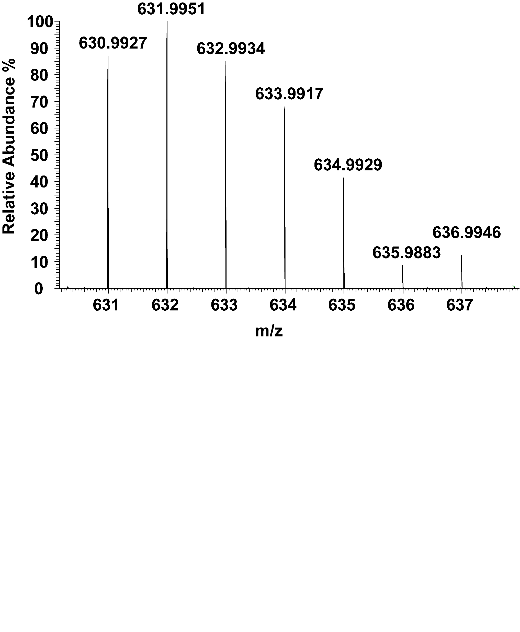

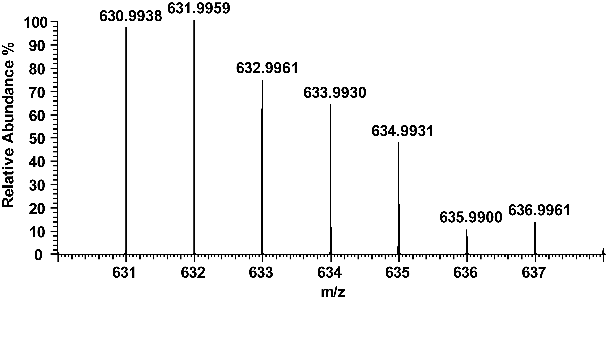


**Fig. S3.** The ESI-HRMS spectrum of **I** (left: found MW; right: calculated MW).

***
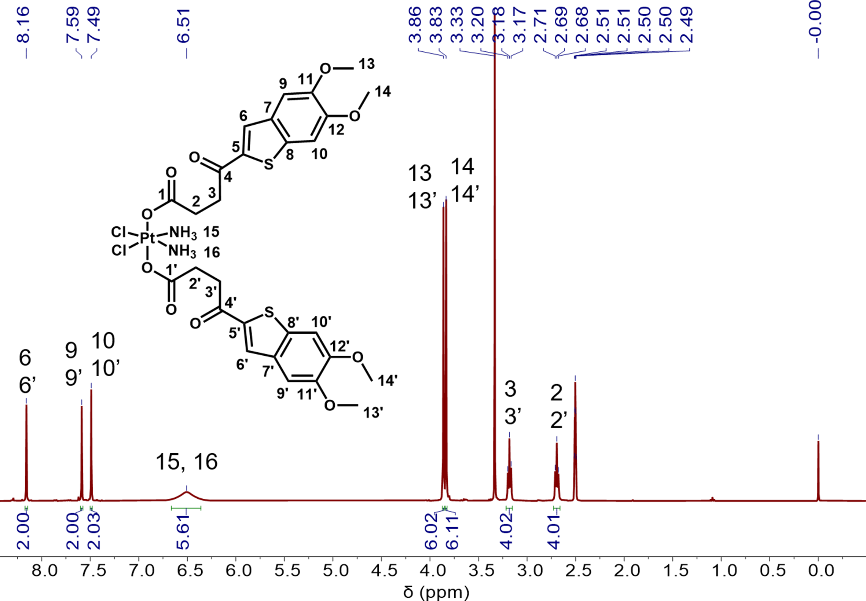
***

**Fig. S4.** The ^1^H-NMR spectrum of **II**.

***
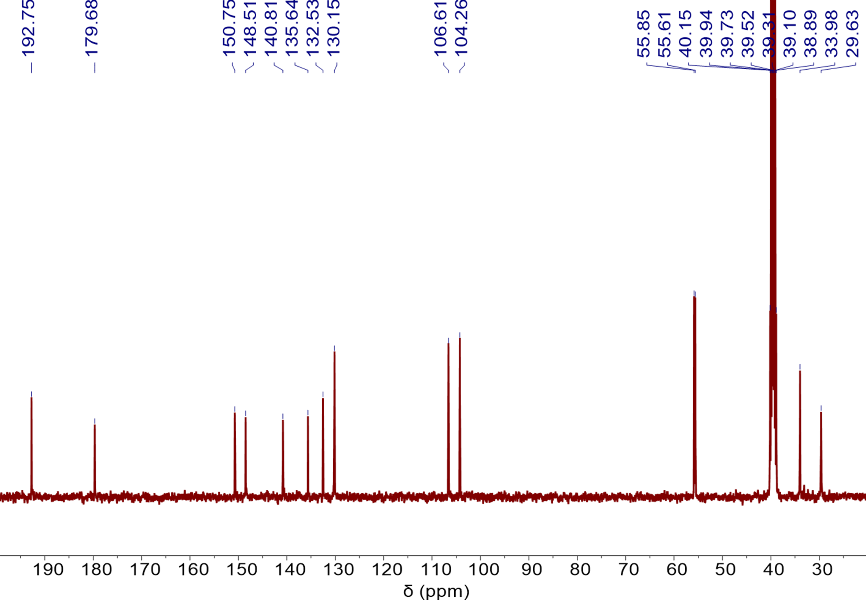
***

**Fig. S5.** The ^13^C{^1^H}-NMR spectrum of **II**.


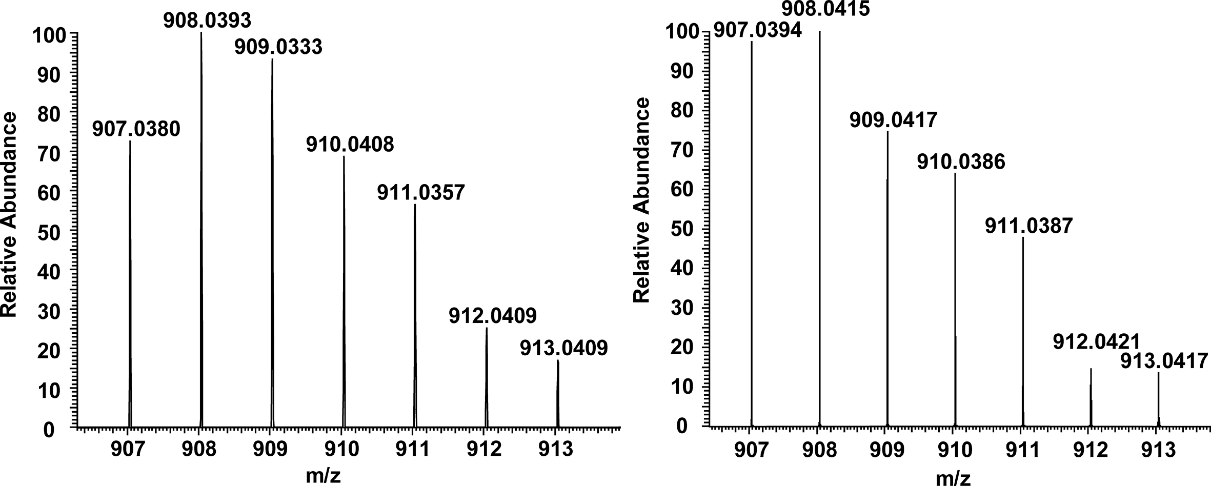


**Fig. S6.** The ESI- HR MS spectrum of **II** (left: found MW; right: calculated MW).


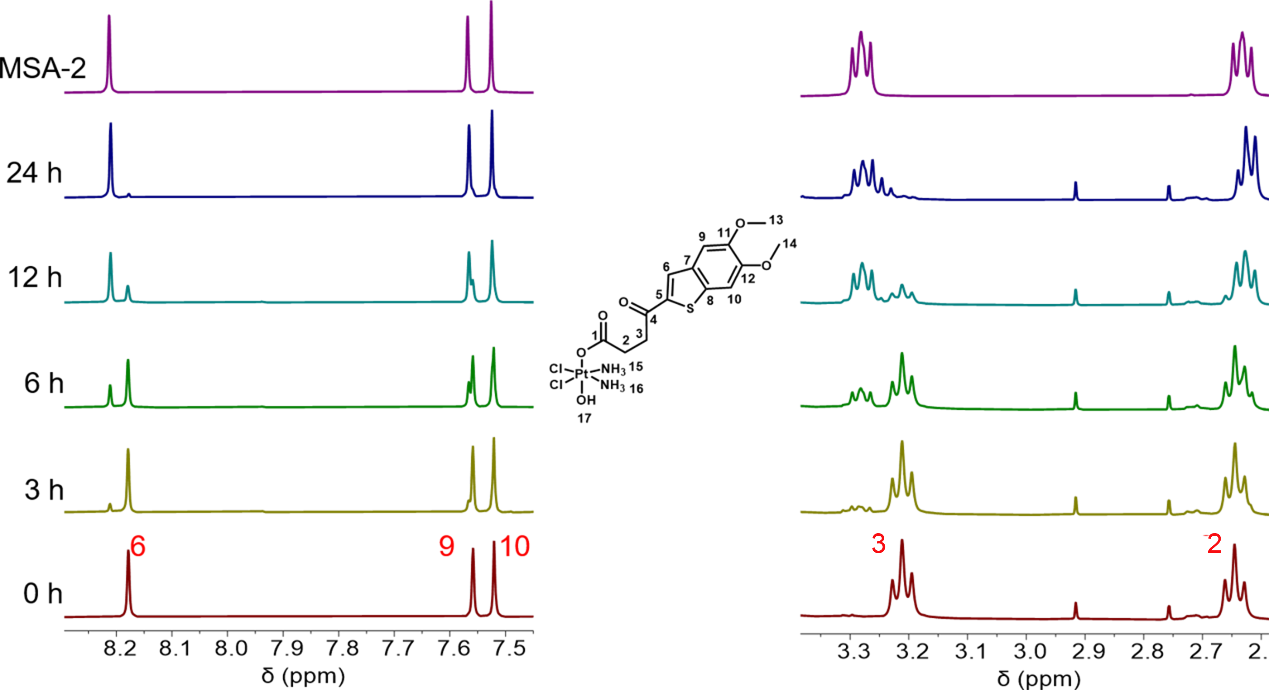


**Fig. S7.** ^1^H-NMR spectra for the reaction of **I** with AsA at 37 ^o^C in the dark. (left: low field; right: high field)


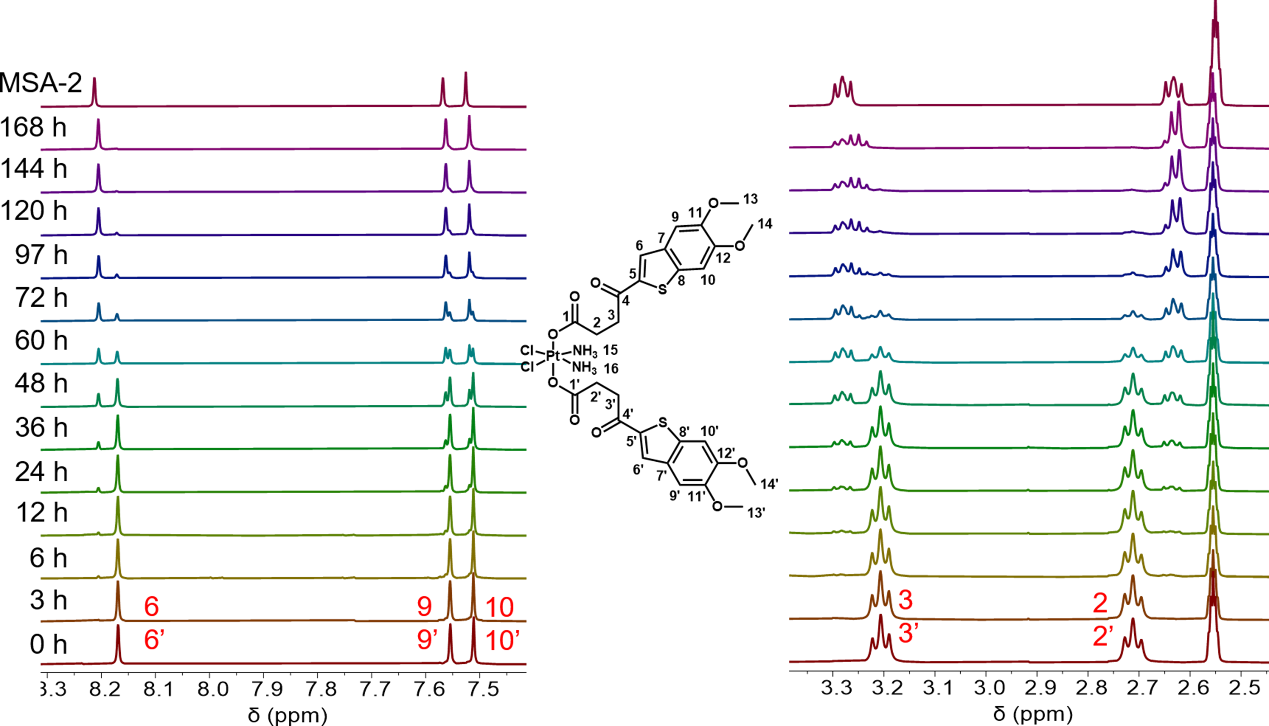


**Fig. S8.** ^1^H-NMR spectra for the reaction of **II** with AsA at 37 ^o^C in the dark. (left: low field; right: high field)


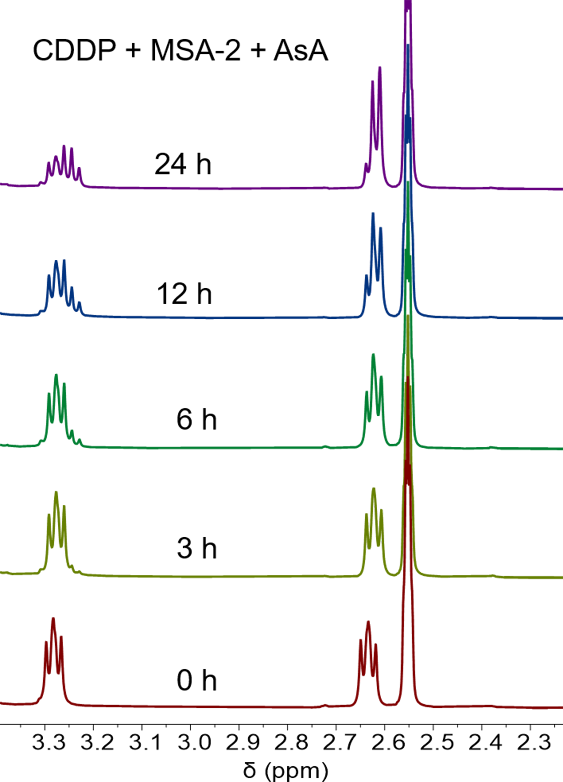


**Fig. S9.** ^1^H-NMR spectra for the mixture of CDDP, MSA-2 and AsA at 37 ^o^C in the dark.


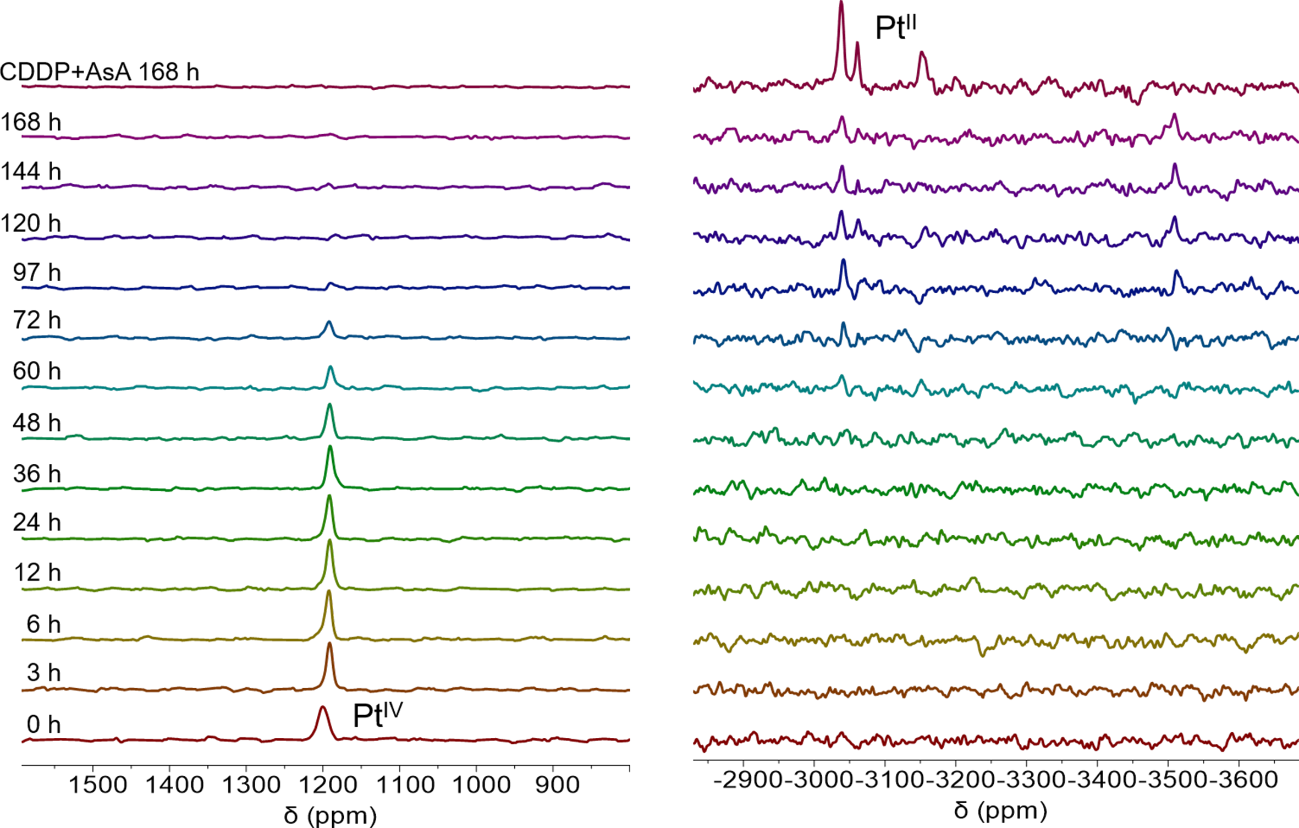


**Fig. S10.** ^195^Pt-NMR spectra for the reaction of **II** with AsA at 37 ^o^C in the dark.

**
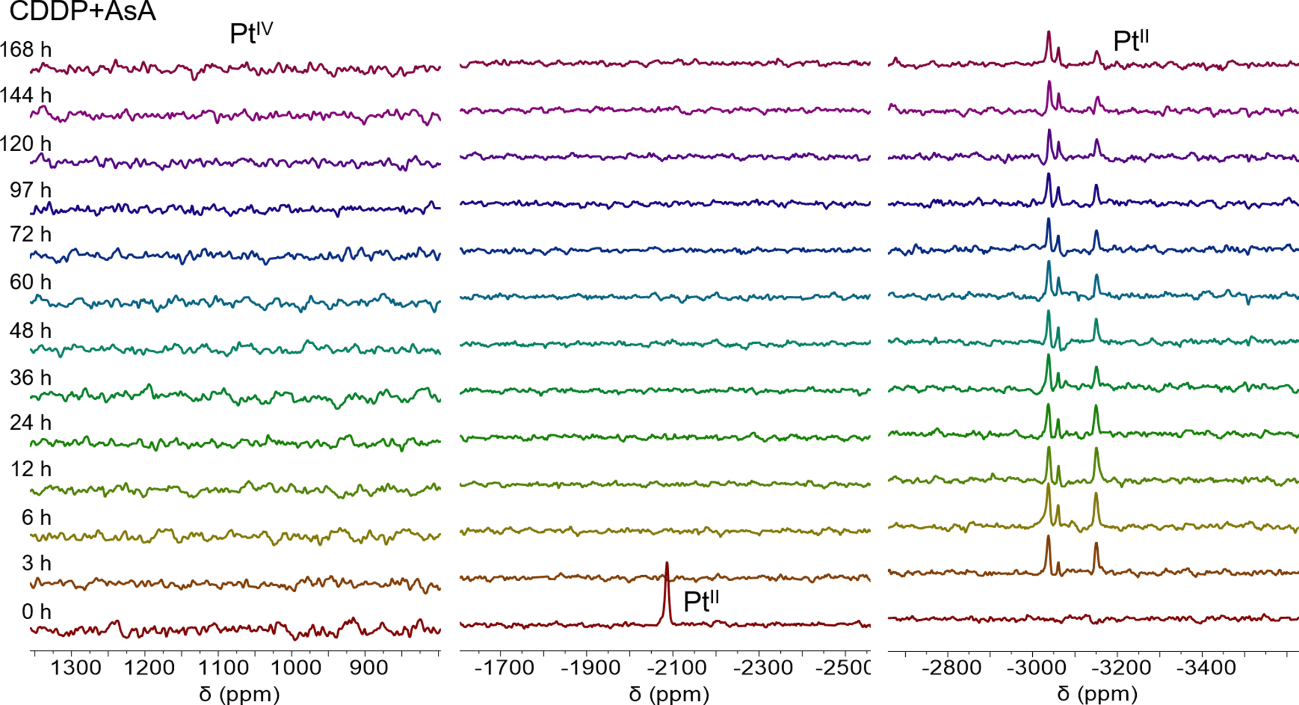
**

**Fig. S11.** ^195^Pt-NMR spectra for the reaction of CDDP with AsA at 37 ^o^C in the dark.

**Table S1.** IC_50_ values (μM) of Pt complexes tested against MCF-7 cell lines, as inferred from 72 h MTT assays.

| **Compounds** | **IC_50_ (μM)** |  |
| --- | --- | --- |
| **I** | 0.3 ± 0.1 | |
| **II** | >64 | |
| CDDP | 5.4 ± 0.3 | |
| CDDP + MSA-2 | 6.7 ± 0.8 | |
| CDDP + 2MSA-2 | 7.7 ± 0.4 | |
| Oxoplatin | 8.2 ± 0.7 | |
| MSA-2 | > 50 | |

**
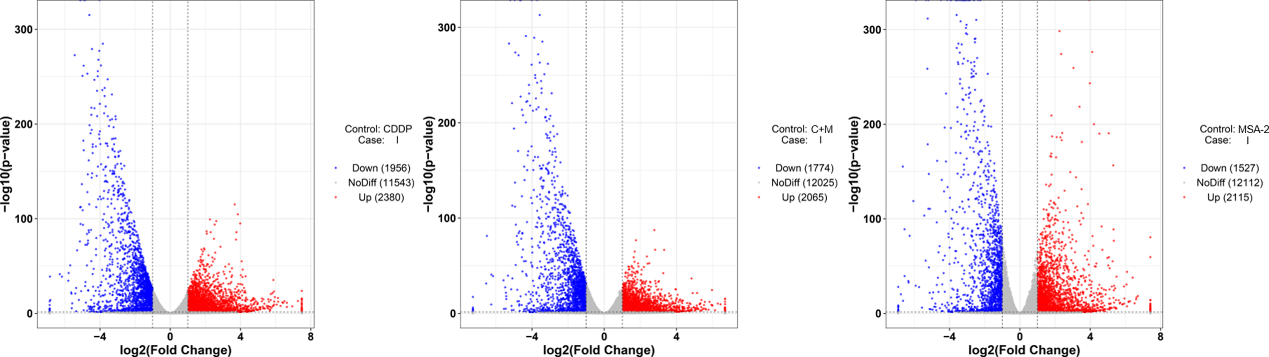
**

**Fig. S12.** Volcano plot of upregulated (red) and downregulated (blue) genes between the **I**-treated and CDDP-, ‘CDDP+MSA-2’-, MSA-2-treated groups. Results with a *p*-value < 0.05 and |log2(fold change)| > 1 were considered significant.


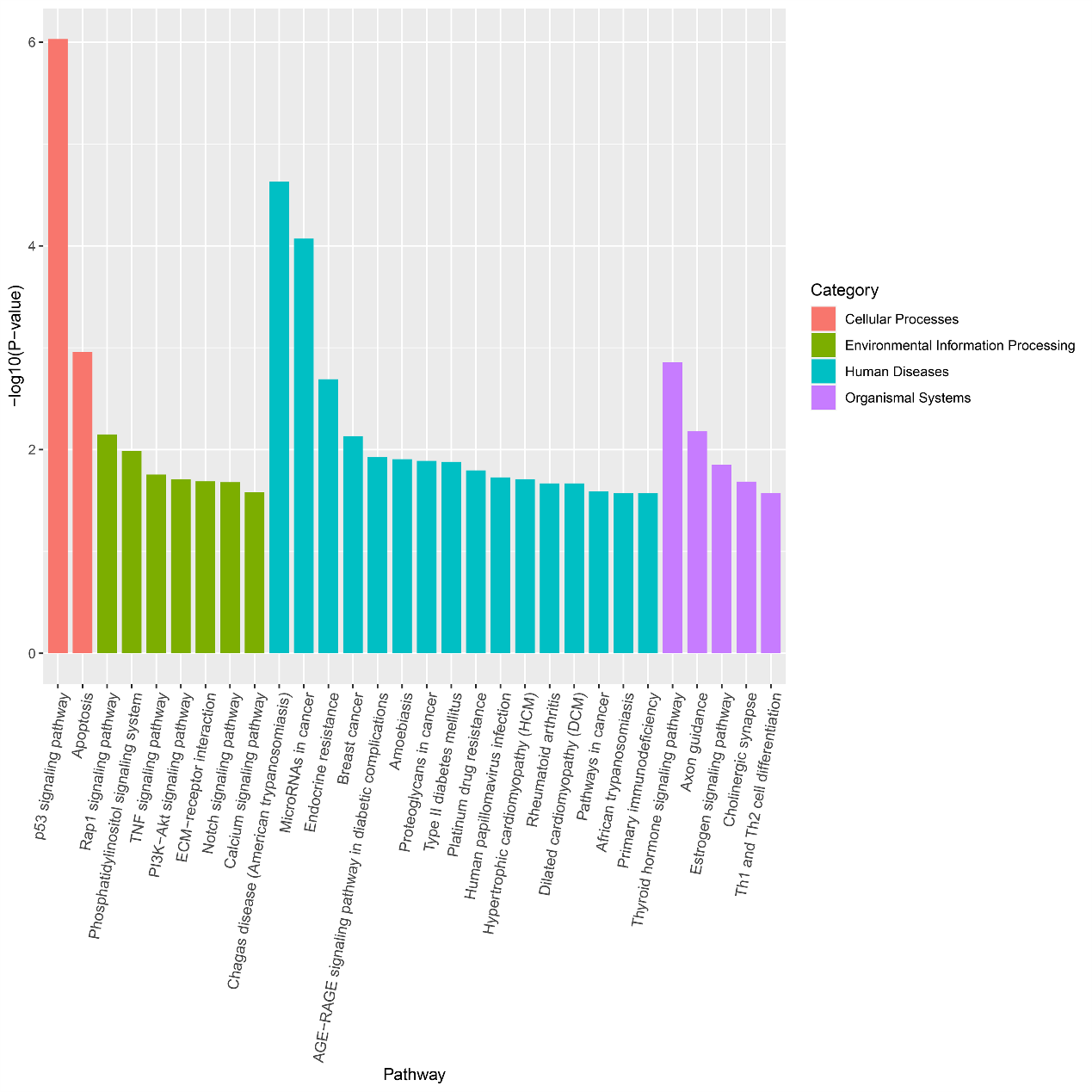


**Fig. S13.** KEGG pathway enrichment analysis of differentially expressed genes in CDDP-treated cells, compared with untreated cells.

**
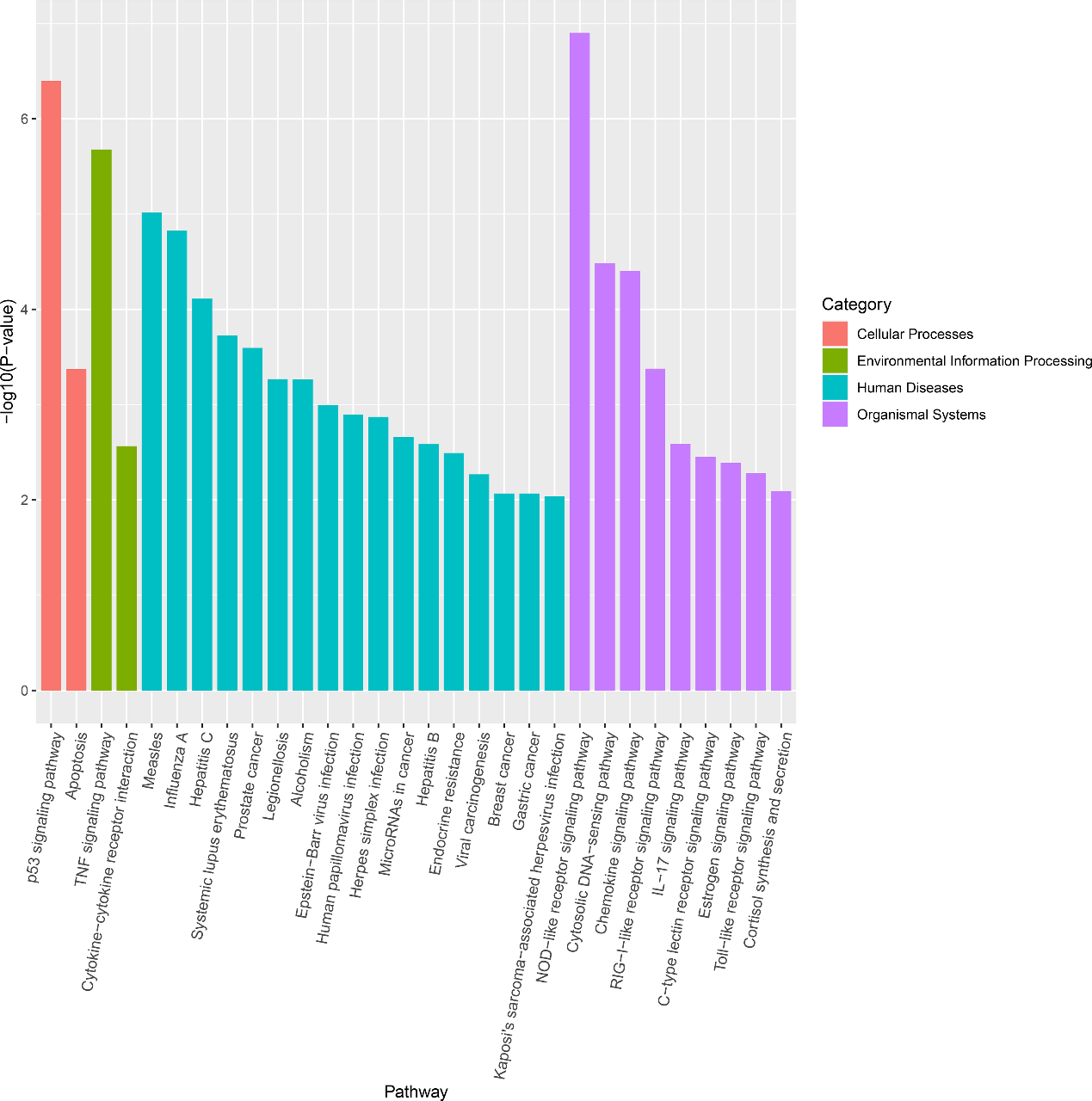
**

**Fig. S14.** KEGG pathway enrichment analysis of differentially expressed genes in ‘CDDP+MSA-2’-treated cells, compared with untreated cells.

**
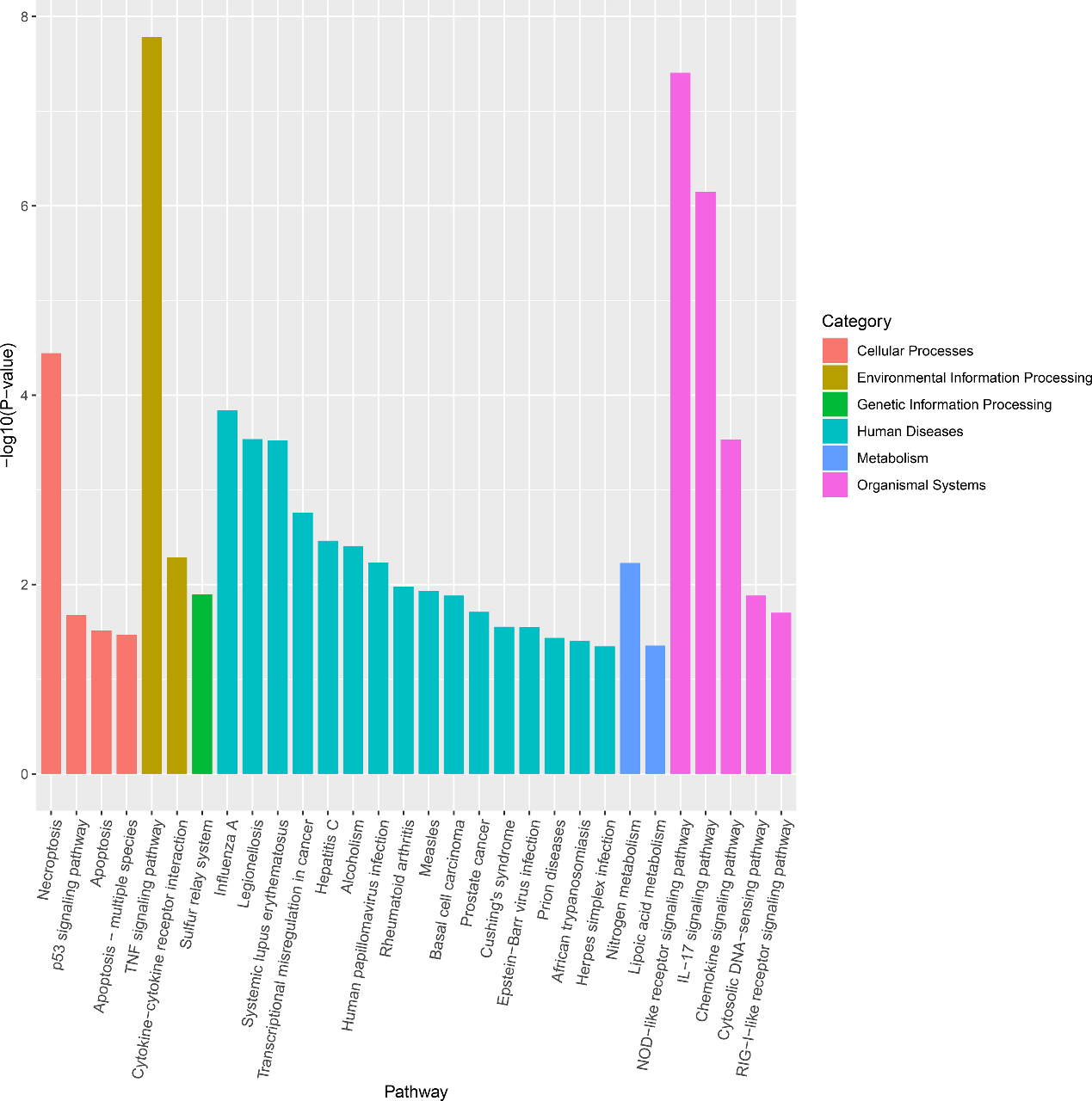
**

**Fig. S15.** KEGG pathway enrichment analysis of differentially expressed genes in MSA-2-treated cells, compared with untreated cells.

**
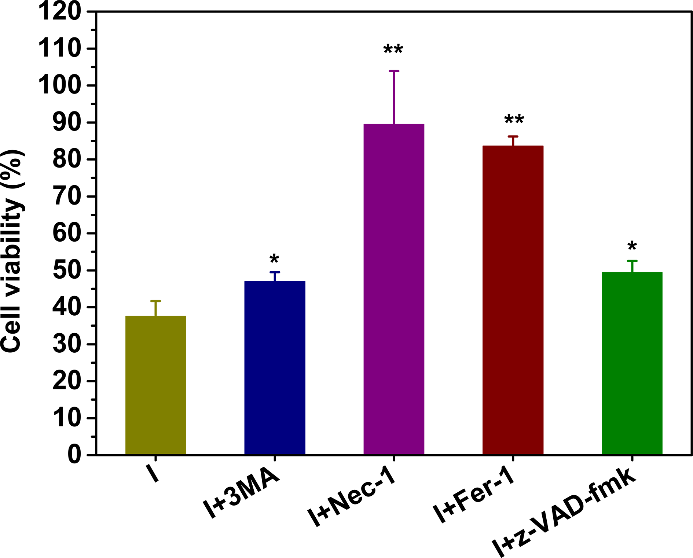
**

**Fig. S16.** Cell viabilities of Pan02 cells determined upon co-incubation (24 h) with **I** (0.2 μM) and different inhibitors. Inhibitor z-VAD-fmk (50 μM), 3-methyladenine (3-MA) (100 μM), ferrostatin-1 (Fer-1) (50 μM), and necrostatin-1 (Nec-1) (50 μM) were administrated 1 h before co-incubation with **I**. NS = not significant, **p* < 0.1, ***p* < 0.01 (versus the **I** group).

**
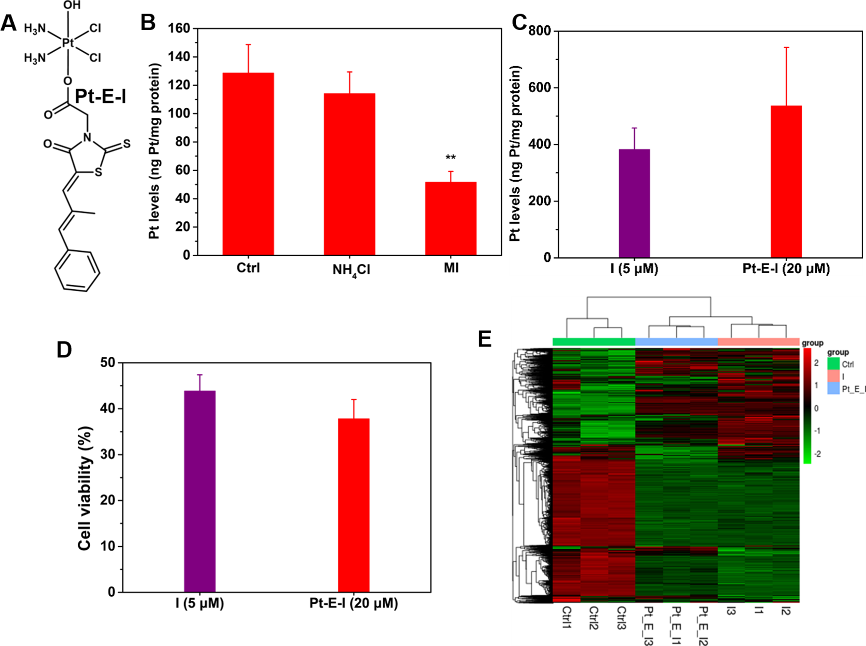
**

**Fig. S17.** (A) The chemical structure of Pt-E-I. (B) Cellular uptake of Pt (ng Pt/mg protein) in Pan02 cells after exposure to Pt-E-I (5 μM) for 6 h. NH_4_Cl: cells preincubated with 50 mM NH_4_Cl; MI: cells preincubated with 50 mM 2-deoxy-D-glucose and 5 μΜ oligomycin. (C) Cellular uptake of Pt (ng Pt/mg protein) in Pan02 cells after exposure to **I** and Pt-E-I (1, 2, 5, 10, 20 μM) for 6 h. (D) The cell viability of Pan02 treated with **I** (5 μM) and Pt-E-I (20 μM). (E) Hierarchical clustering of differentially expressed genes between **I**-treated (5 μM) Pan02 cells, Pt-E-I-treated (20 μM) and nontreated cells.

**
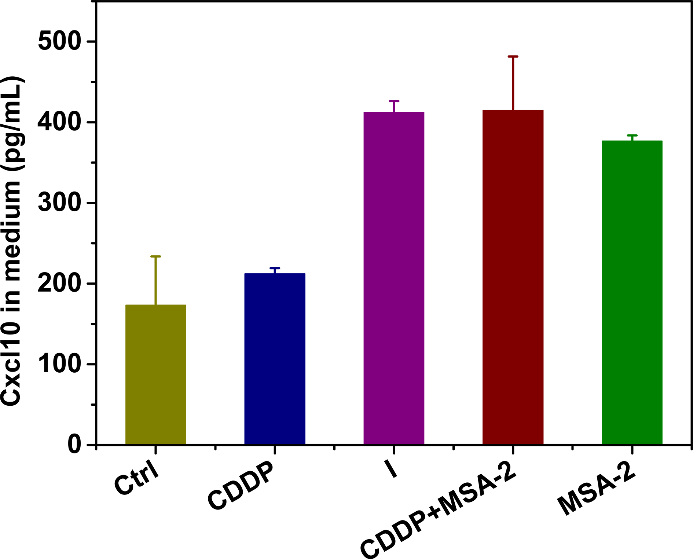
**

**Fig. S18.** Cxcl10 concentrations in the supernatants of Pan02 cell incubated with Pt complexes (5 μM, 24h).


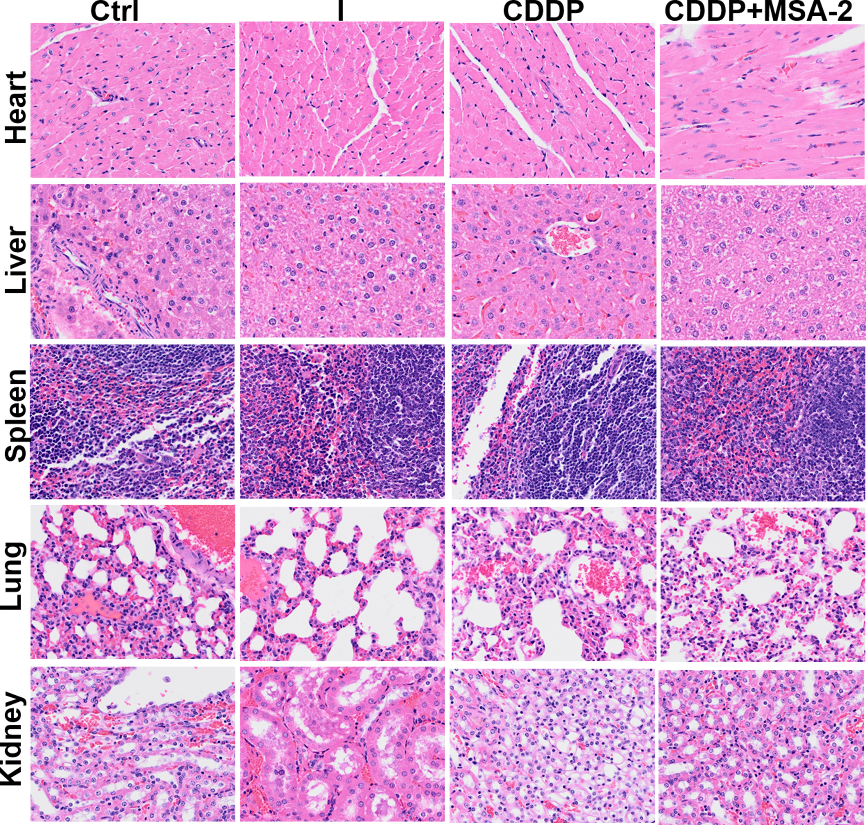


**Fig. S19.** H&E-stained images of heart, liver, spleen, lung and kidney sections collected from mice after treatment.


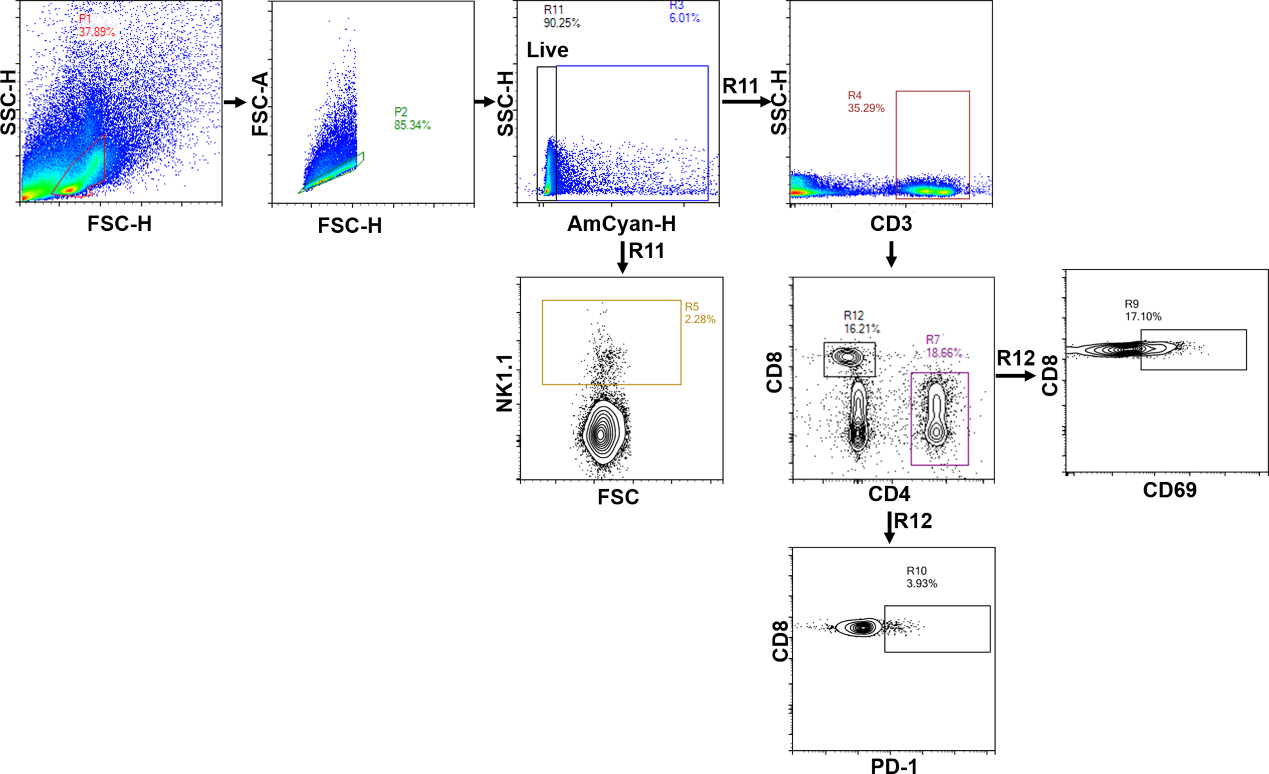


**Fig. S20.** Flow cytometric gating strategies for Pan02 tumor tissues after treatment, related to Figs. 4, D, E, F and G, Fig. S21.


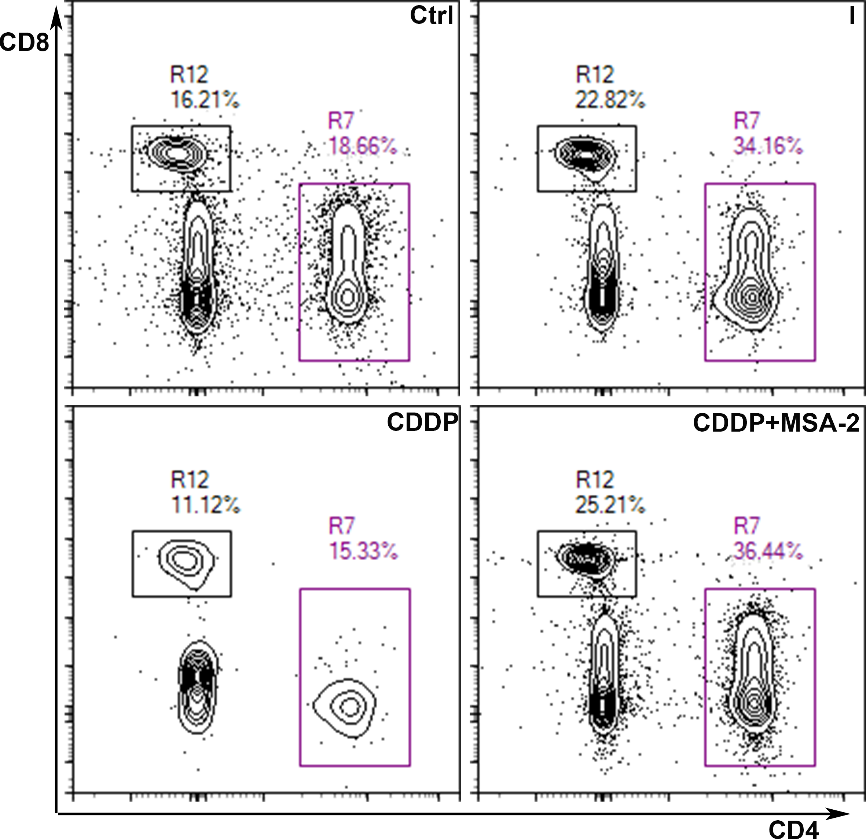


**Fig. S21.** Flow cytometric analysis of CD4^+^ and CD8^+^ T cells in Pan02 tumor tissues among different treatment groups.


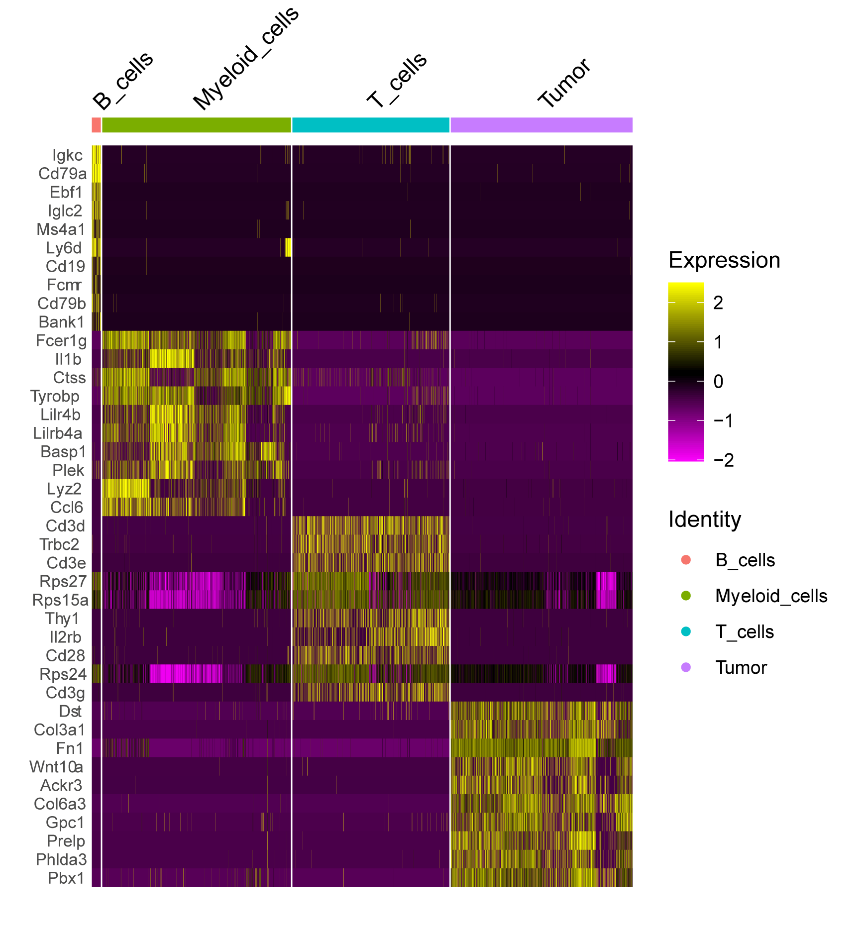


**Fig. S22.** Heatmap illustration of immune- and tumor-cell clustering signature genes. The colors on top of the map indicate the cell clusters. The ten marker genes used to identify each cluster are listed alongside the cluster.

**
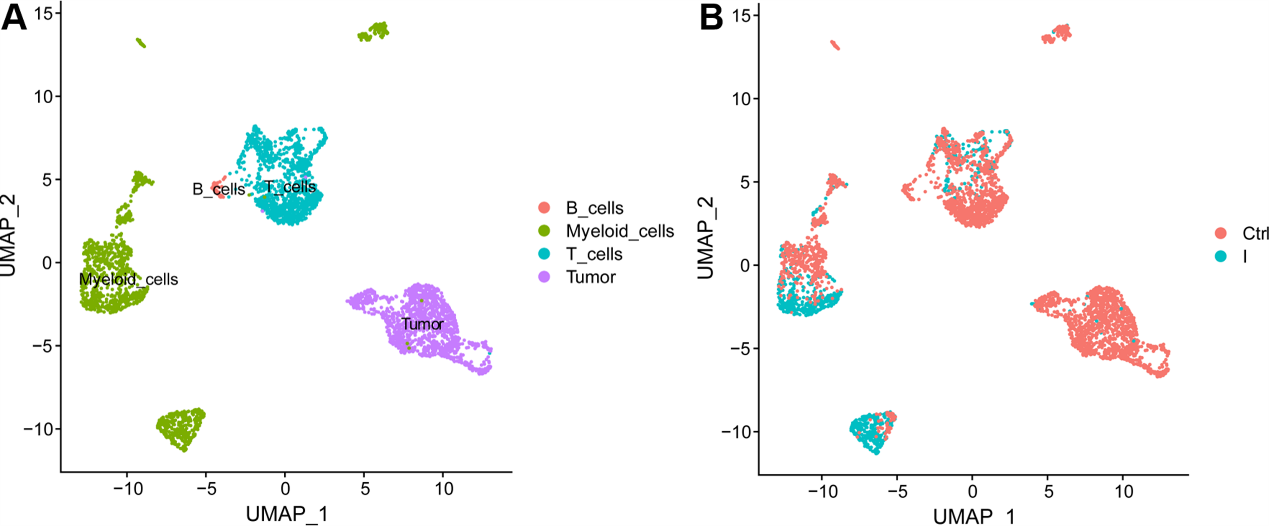
** **Fig. S23.** Cell annotation of the scRNA-seq analysis of Pan02 tumors. (A) Unsupervised clustering of tumor and immune cells derived from tumors excised from untreated mice and pooled. (B) Clusters in (A) colored by the control and **I**-treated mice.


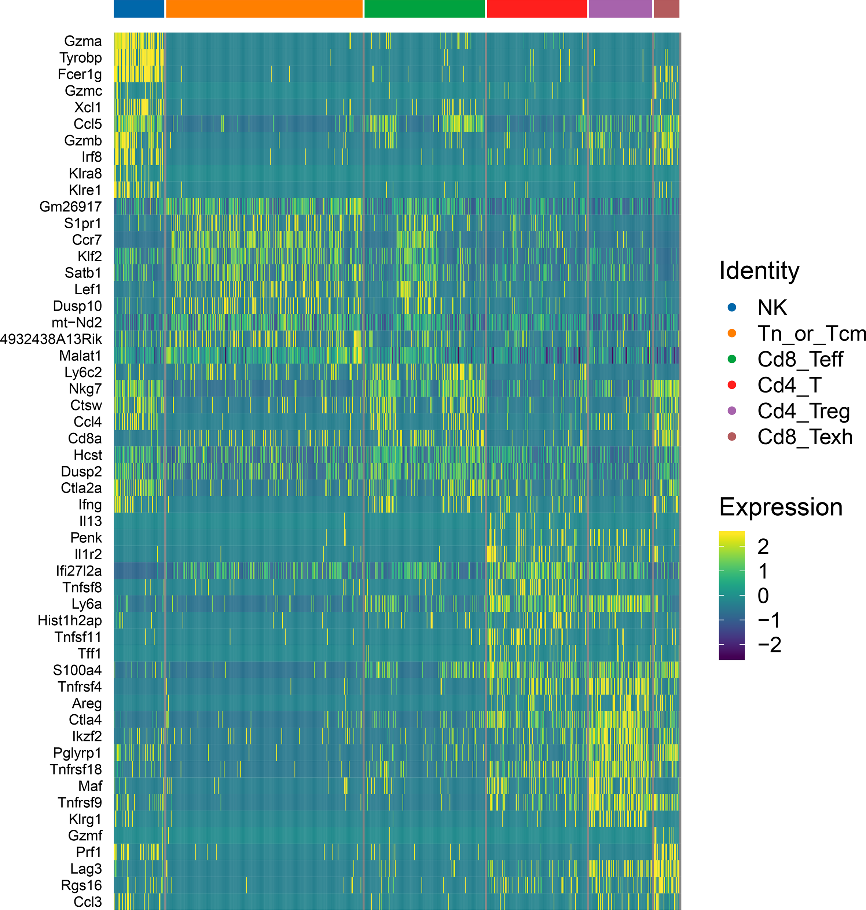


**Fig. S24. Heatmap illustration of immune-cell clusters with unique signature genes.** The colors on top of the map indicate the cell clusters. The ten marker genes used for each cluster are listed alongside the cluster.


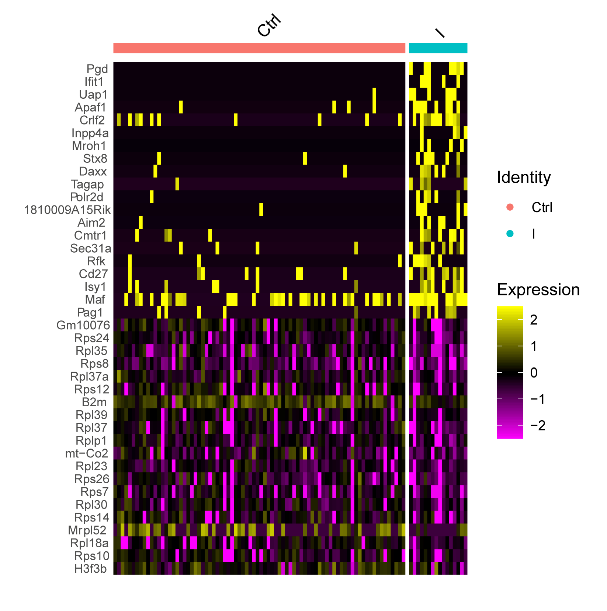

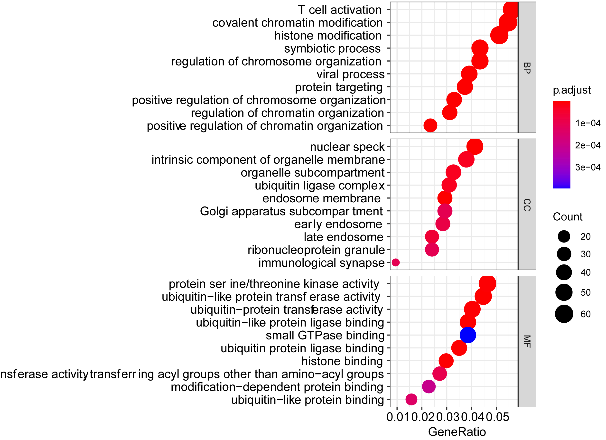


**Fig. S25.** Heat map showing the relative expression of transcripts in Treg cells from the control and I-treated groups(left); GO analysis of upregulated transcripts in Treg cells from the I-treated group compared with those from the control groups (right).

**Table S2.** CD8 effector T cells showed enrichment of genes in virus-associated pathways by GO analysis.

| Description | gene ID |
| --- | --- |
| response to virus | Ifitm3/Tnf/Exosc4/Rsad2/Oas3/Ifng/Isg15/Stat2/Bcl3/Ccl5/Dtx3l/Cd37/Isg20/Ifngr1/Trim56/Tagap/Serinc3/Zbp1/Ddx58/Ifitm2/Slfn8 |
| defense response to virus | Ifitm3/Exosc4/Rsad2/Oas3/Ifng/Isg15/Stat2/Ccl5/Dtx3l/Cd37/Isg20/Ifngr1/Trim56/Tagap/Serinc3/Zbp1/Ddx58/Ifitm2/Slfn8 |


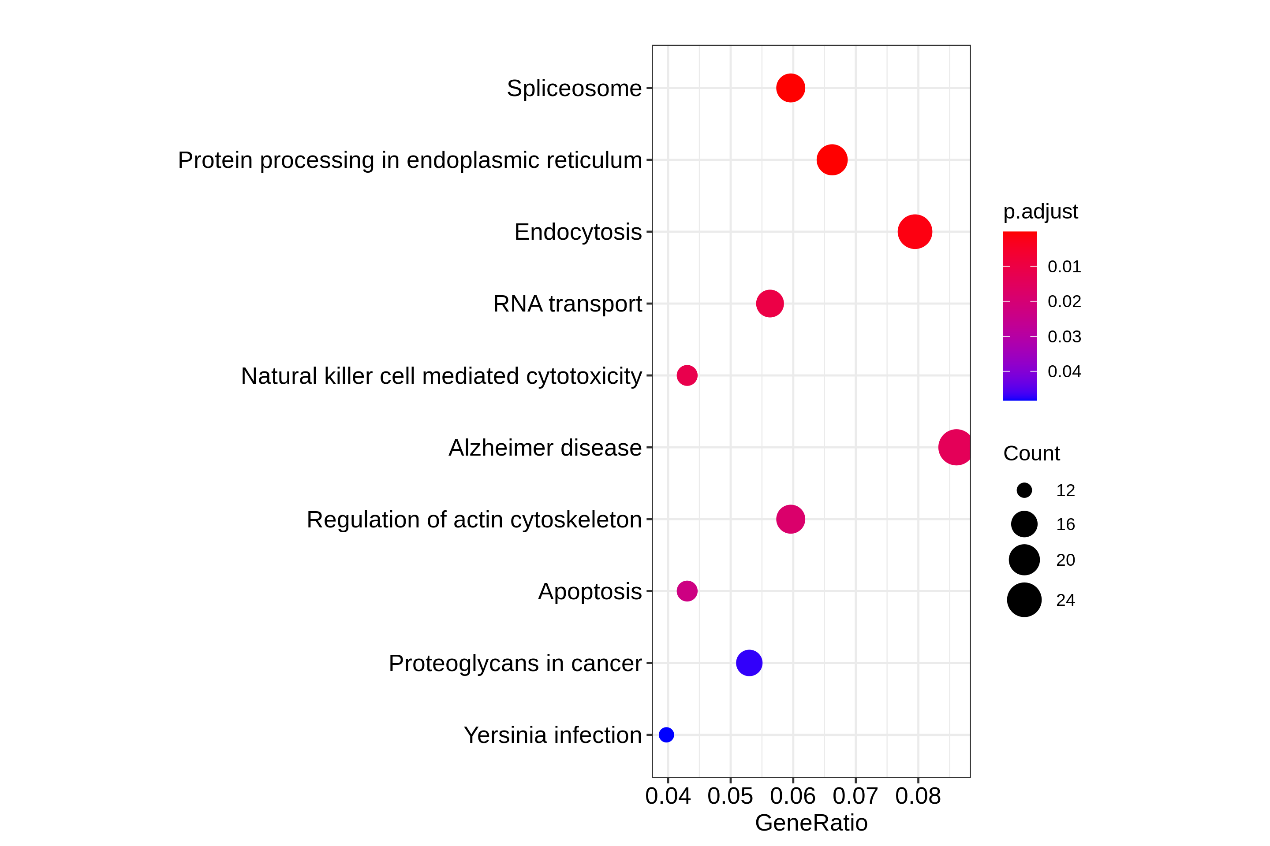


**Fig. S26.** KEGG analysis of upregulated transcripts in NK cells from the **I**-treated Pan02 tumors, compared with the control group.


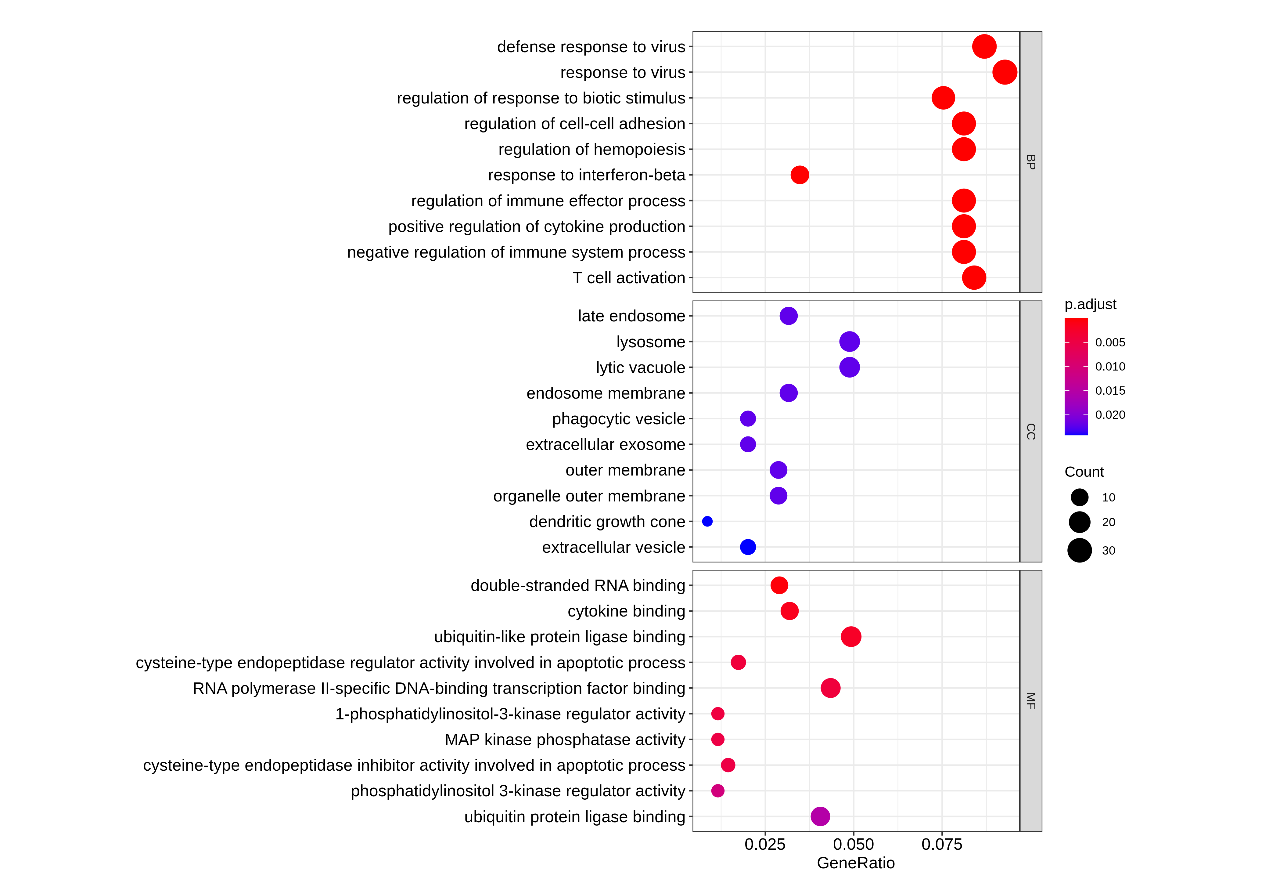


**Fig. S27.** GO analysis of upregulated transcripts in DCs from the **I**-treated Pan02 tumors, compared with the control group.

**
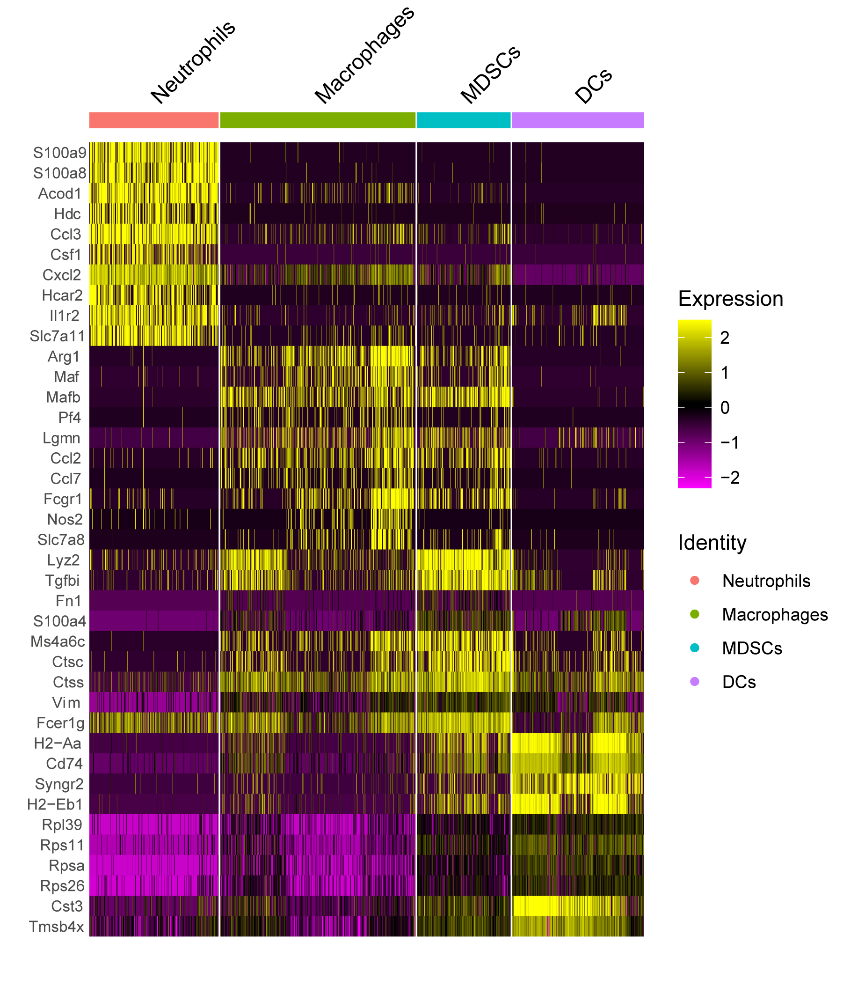
**

**Fig. S28.** Heatmap illustration of myeloid-cell clusters with unique signature genes. The colors on top of the map indicate the cell clusters. The ten marker genes used for each cluster are listed alongside the cluster.


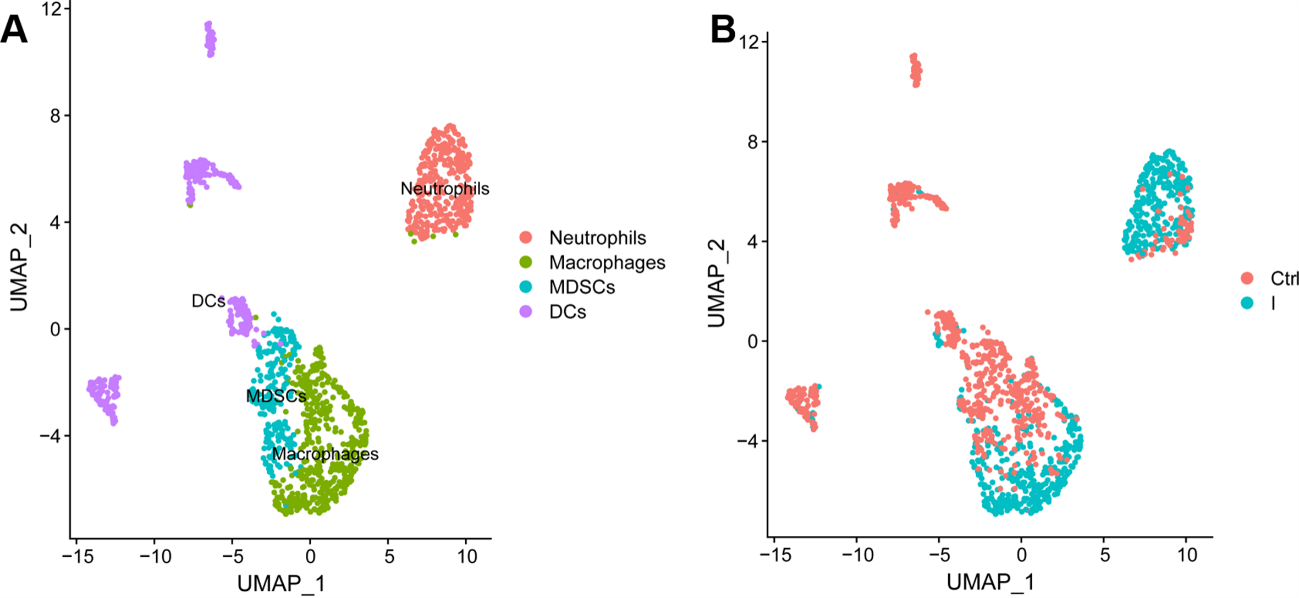


**Fig. S29.** Cell annotation of the scRNA-seq analysis of Pan02 tumors. (A) Unsupervised clustering of myeloid cells derived from tumors excised from untreated mice and pooled. (B) Clusters in (A) colored by the control and **I**-treated mice.


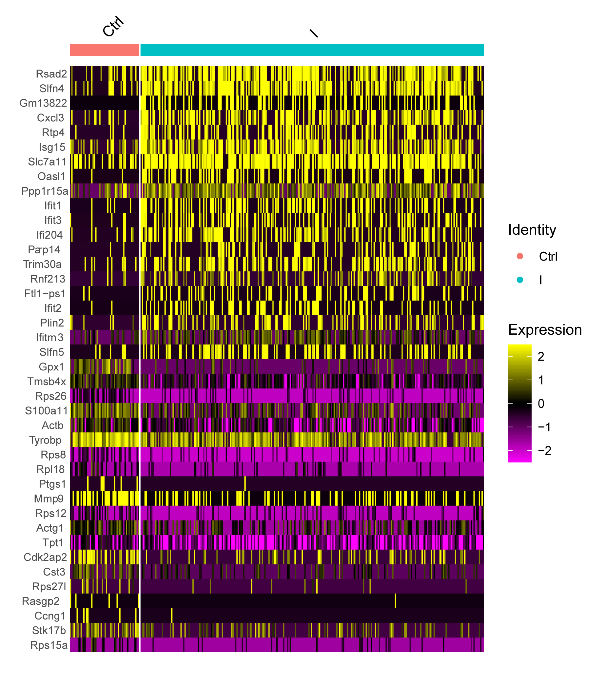

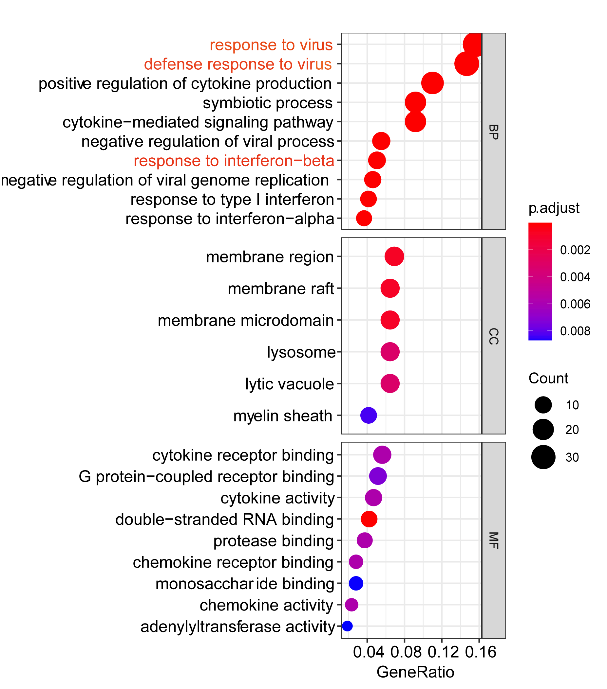


**Fig. S30.** Heat map showing the relative expression of transcripts in Neutrophils from the control and **I**-treated groups(left); GO analysis of upregulated transcripts in Neutrophils from the **I**-treated group compared with those from the control groups (right).


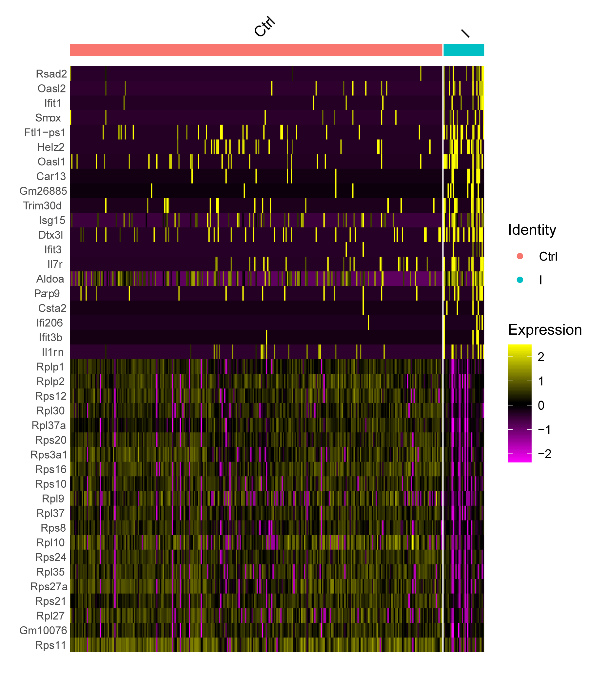

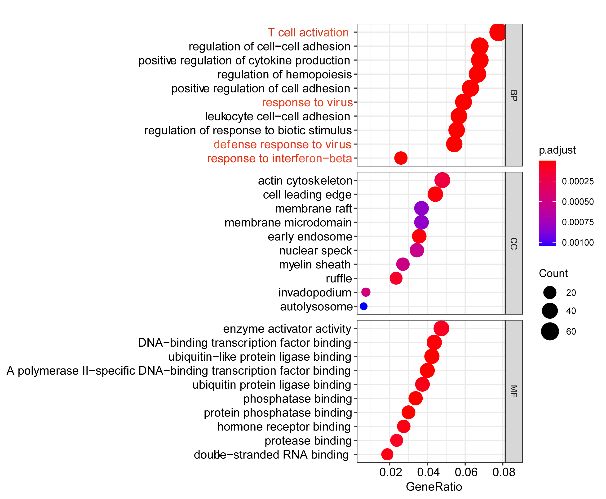


**Fig. S31.** Heat map showing the relative expression of transcripts in DCs from the control and **I**-treated groups(left); GO analysis of upregulated transcripts in DCs from the **I**-treated group compared with those from the control groups (right).


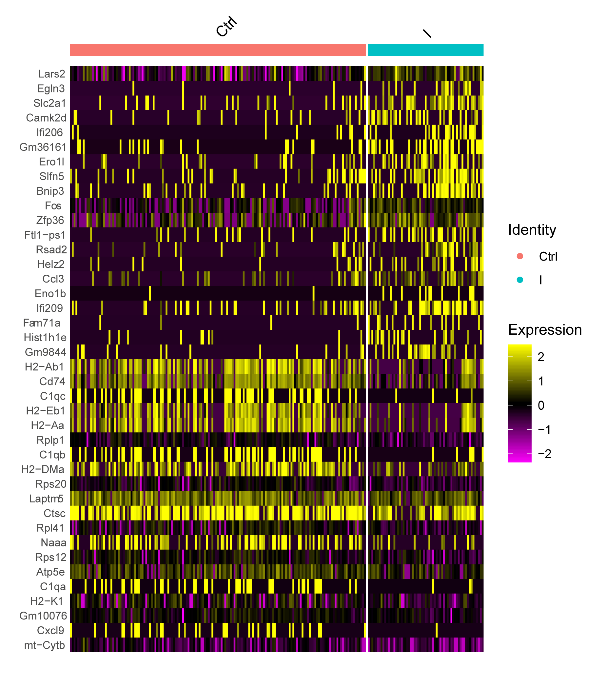

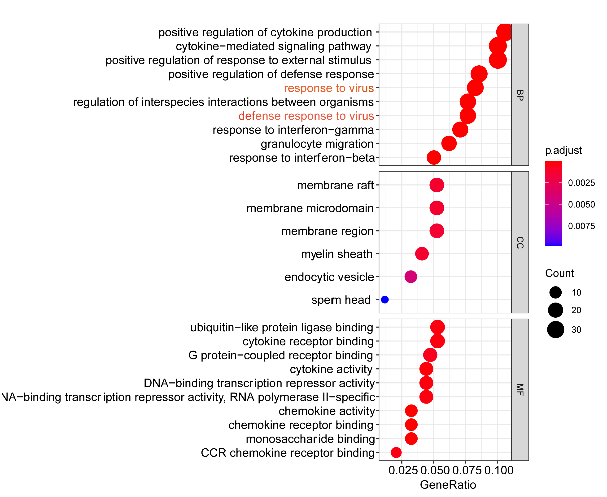


**Fig. S32.** Heat map showing the relative expression of transcripts in MDSCs from the control and **I**-treated groups(left); GO analysis of upregulated transcripts in MDSCs from the **I**-treated group compared with those from the control groups (right).


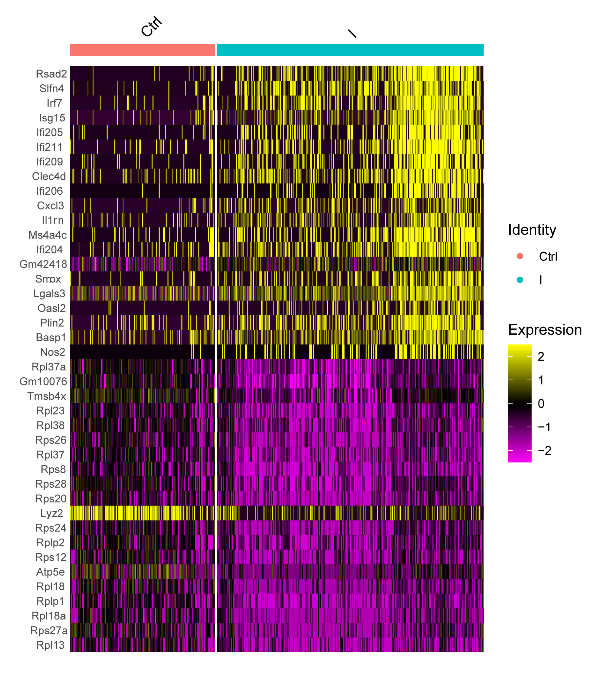

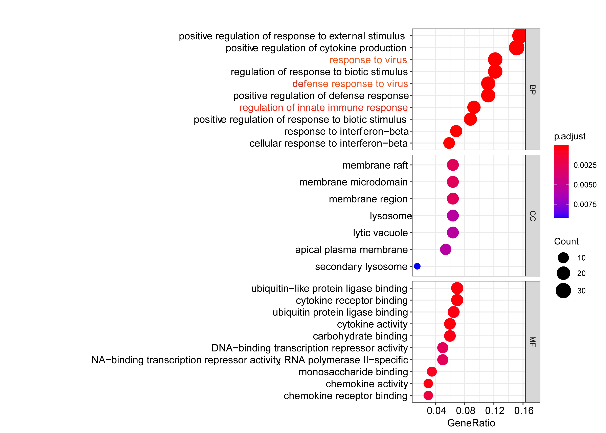


**Fig. S33.** Heat map showing the relative expression of transcripts in Macrophages from the control and **I**-treated groups(left); GO analysis of upregulated transcripts in Macrophages from the **I**-treated group compared with those from the control groups (right).

**Reference**

1. Anders, S, Pyl, PT, Huber, W. HTSeq--a Python framework to work with high-throughput sequencing data. *Bioinformatics*. 2015; **31**(2): 166-169.

2. Simon Anders, WH. Differential expression analysis for sequence count data. *Genome Biology*. 2010; **11**: 1-12.

3. Hu, J, Chen, Z, Bao, L*, et al.* Single-Cell Transcriptome Analysis Reveals Intratumoral Heterogeneity in ccRCC, which Results in Different Clinical Outcomes. *Mol. Ther*. 2020; **28**(7): 1658-1672.

4. Chen, Z, Zhou, L, Liu, L*, et al.* Single-cell RNA sequencing highlights the role of inflammatory cancer-associated fibroblasts in bladder urothelial carcinoma. *Nat. Commun*. 2020; **11**(1): 5077.

5. Yu, G, Wang, LG, Han, Y*, et al.* clusterProfiler: an R package for comparing biological themes among gene clusters. *OMICS*. 2012; **16**(5): 284-287.
